# Potential Anti-Acetylcholinesterase Activity of *Cassia timorensis* DC.

**DOI:** 10.3390/molecules25194545

**Published:** 2020-10-04

**Authors:** Nurul Amira Nurul Azman, Maram B. Alhawarri, Mira Syahfriena Amir Rawa, Roza Dianita, Amirah Mohd Gazzali, Toshihiko Nogawa, Habibah A. Wahab

**Affiliations:** 1 School of Pharmaceutical Sciences, Universiti Sains Malaysia, 11800 Minden, Malaysia; nurulamira.nazman@gmail.com (N.A.N.A.); maram.alhawarri@gmail.com (M.B.A.); mirasyahfriena@gmail.com (M.S.A.R.); rozadianita@gmail.com (R.D.); 2 USM-RIKEN Centre for Aging Science (URICAS), Universiti Sains Malaysia, 11800 Minden, Malaysia; nogawat@riken.jp; 3 Chemical Biology Research Group, RIKEN Centre for Sustainable Resource Science, 2-1 Hirosawa, Wako, Saitama 351-0198, Japan

**Keywords:** *Cassia timorensis*, *Senna timoriensis*, benzenepropanoic acid, 9,12,15-octadecatrienoic acid, 3-methoxyquercetin, acetylcholinesterase, molecular docking

## Abstract

Seventeen methanol extracts from different plant parts of five different *Cassia* species, including *C. timorensis*, *C. grandis*, *C. fistula*, *C. spectabilis*, and *C. alata* were screened against acetylcholinesterase (AChE). *C. timorensis* extracts were found to exhibit the highest inhibition towards AChE whereby the leaf, stem, and flower methanol extracts showed 94–97% inhibition. As far as we are aware, *C. timorensis* is one of the least explored *Cassia* spp. for bioactivity. Further fractionation led to the identification of six compounds, isolated for the first time from *C. timorensis*: 3-methoxyquercetin (**1**), benzenepropanoic acid (**2**), 9,12,15-octadecatrienoic acid (**3**), β-sitosterol (**4**), stigmasterol (**5**), and 1-octadecanol (**6**). Compound **1** showed moderate inhibition towards AChE (IC_50_: 83.71 μM), while the other compounds exhibited poor to slightly moderate AChE inhibitory activity. Molecular docking revealed that the methoxy substitution of **1** formed a hydrogen bond with TYR121 at the peripheral anionic site (PAS) and the hydroxyl group at C5 formed a covalent hydrogen bond with ASP72. Additionally, the OH group at the C3′ position formed an interaction with the protein at the acyl pocket (PHE288). This possibly explains the activity of **1** in blocking the entry of acetylcholine (ACh, the neurotransmitter), thus impeding the hydrolysis of ACh.

## 1. Introduction

*Cassia* is a huge genus of flowering plants belongs to the Fabaceae family which comprised of more than 500 species that are diverse in herbs, shrubs, and trees [1]. This genus is widely distributed in tropical countries such as the South-East Asia, tropical America and African regions. Taxonomically, species of the *Cassia* genus are easily identified due to their bright yellow, pink, and white flowers, along with the presence of legume fruits. Many species of *Cassia* have medicinal values and they might differ from one to another depending on their phytochemical constituents [2]. For most known *Cassia* species, the leaves, stems, pods, and seeds have been shown to possess different pharmacological values [3,4]. Among the known ethnopharmacological applications of *Cassia* spp. are the treatment of skin diseases such as eczema, ringworm, scabies, and leprosy [5,6,7]. Reported in vitro studies also showed their possible use as a treatment for human brain disorders*,* such as epilepsy, migraine, and hysteria [8,9].

Alzheimer’s disease (AD) has become a major concern worldwide as no cure has been found yet. To date, three clinical drugs have been approved by the US Food and Drug Administration (FDA), which are all cholinesterase inhibitors; donepezil, galanthamine, and rivastigmine [10]. AD is classified as a neurodegenerative disorder. It is characterized by consistent loss of neurons in the cognitive system, the appearance of amyloid plaques, intercellular hyper-phosphorylated neurofibrillary tangles (NFTs), and an inadequate level of acetylcholine (ACh), a neurotransmitter in the brain [11]. The acetylcholinesterase enzyme (AChE) has an important role in the treatment of AD, where impediment of its activity (hydrolyzing the acetylcholine (ACh) neurotransmitter into choline and acetate) will help to maintain the longevity of the neurotransmitter in the cerebral cortex [12,13]. ACh transports signals within neurons and it significantly contributes to memory and learning. Over-catalysis of ACh by AChE will decrease the level of ACh in the brain specifically in the nucleus basalis of Meynert and eventually will worsen the AD symptoms [14]. At present, several natural compounds have been tested for their ability to inhibit AChE and among them are terpenes, coumarins, xanthones, isoquinolines, piperidine alkaloids [11,15], anthraquinones [16] and flavonoids [17]. Thus far, *Cassia* species have been reported to be abundant in anthraquinones and flavonoids, which are responsible for their ethno-pharmaceutical values [18,19,20]. Of the known plants in the genus *Cassia*, *Cassia timorensis* is among the least explored. The pharmacological values of this particular species are yet to be uncovered as evidenced by the lack of information in the literature. *Cassia timorensis* (DC.) H. S. Irwin & Barneby is currently considered as a synonym of *Senna timoriensis* (DC.) H. S. Irwin & Barneby as recorded in “The Plant List” website [21]. To date, only one compound has been reported in the literature that was isolated from *C. timorensis*. This compound known as barakol was isolated from the aqueous acetic acid extract of the leaves [22]. Barakol was also previously isolated from *Cassia siamea* and reported to have sedative and anxiolytic effects [23,24]. Here, we present the results of AChE inhibition of selected *Cassia* spp.*,* which led us towards the phytochemical investigation of *C. timorensis*. The isolated compounds were also tested for their anti-AChE activity through in vitro and in silico studies in our quest to further understand the inhibition mechanism at the molecular level.

## 2. Results and Discussion

### 2.1. Inhibitory Activity of Acetylcholinesterase by Cassia spp.

The inhibitory activity of the methanol extracts of five *Cassia* spp., i.e., *C. timorensis, C. grandis, C. fistula, C. spectabilis* and *C. alata*, was carried out at 0.2 mg/mL and classed according to poor, moderate or good inhibitions (poor: 5–25%; moderate: 25–50%; good: 50–100%); the results are presented in Table 1. From the 17 extracts, 6 showed positive results with the percentage of inhibition greater than 50% (good inhibition). *C. timorensis* methanol extracts showed good inhibitory activity, exhibiting the highest inhibitory percentage for extracts from leaves, stems, and flowers at 94.69%, 97.16%, and 96.94%, respectively. *C. grandis* leaf and stem extracts similarly displayed good AChE inhibitory activity with 86.09% and 91.76%, respectively. Apart from these two plants, only the stems crude extract of *C. fistula* showed a good inhibitory activity at 88.66%. Methanolic extracts that showed inhibition at <50%, or no inhibition towards AChE, were considered as moderate and poor inhibitors. Following these observations, we selected *C. timorensis* for further study.

The aqueous, butanol, and ethyl acetate fractions of *C. timorensis* leaf extract (Table 2) exhibited good inhibitory activity with 98.80%, 91.83%, and 98.98% inhibition at 0.2 mg/mL, respectively. Likewise, hexane, ethyl acetate, and butanol fractions from *C. timorensis* flowers also exhibited good inhibitory activity with 92.35%, 95.98%, and 98.16%, respectively. On the other hand, for the stems, the ethyl acetate fraction only showed 30.84% inhibitory activity, while the butanol and aqueous fractions showed good activity with 94.92 and 84.35% inhibition, respectively. We postulated that ethyl acetate might yield semi-polar compounds, which might be able to cross the blood-brain barrier better than the polar compounds as suggested by Li and co-workers [25]. Therefore, we selected the ethyl acetate fractions of the leaves and flowers of *C. timorensis* for IC_50_ determination. The stem was not selected as its ethyl acetate did not show encouraging inhibitory activity against AChE. The IC_50_ of both the leaves and flowers’ ethyl acetate fractions seemed promising (IC_50_ of 19.21 ± 4.16 µg/mL (leaves) and 12.75 ± 6.28 µg/mL (flowers)). This encouraged us to further attempt to isolate compounds from the ethyl acetate fractions of this *Cassia* spp. In addition, the hexane fraction of the flowers also demonstrated good inhibition towards AChE; thus, we also selected the hexane fractions of these plant parts in our isolation study (IC_50_ of the hexane fraction of the flower is 12.98 ± 0.95 µg/mL).

### 2.2. Identification of Isolated Compounds

Yellow needles of compound **1** were isolated from the ethyl acetate fraction of *C. timorensis* leaves and characterized as 3-methoxyquercetin. Compound **1** or 3-methoxyquercetin, is a flavonol with a TLC retention factor (Rf) value of 0.62 (Hex/EtAc: 2:8). The ^1^H- and ^13^C-NMR spectra showed characteristic signals for flavonol skeletons with a methoxy group substitution at the C3 position. Compounds **2** and **3** appeared as yellowish oil and were isolated from the hexane fraction of *C. timorensis* leaf extract. Compound **2,** characterized as benzenepropanoic acid, was recently isolated from *Trigonella foenum* (Fabaceae) and demonstrated to have anti-cancer potential through in vitro and in vivo studies [26]. To the best of our knowledge, there was no report on the isolation of this compound from the genus *Cassia*, although it has been reported to be present in *C. fistula* seeds as identified from GC-MS [27]. Compound **3** was characterized as 9,12,15-octadecatrienoic acid, also known as linolenic acid. Linolenic acid is a fatty acid that can be found abundantly in most of the plants in the genus *Cassia* [28].

On the other hand, white crystalline needles, with Rf values of 0.68 (EtAc/Hex: 3:7) were isolated. On the basis of spectral data analysis as well as by comparing the spectral data to literature data, compounds **4** and **5** have been established as a mixture of β-sitosterol and stigmasterol. β-sitosterol and stigmasterol are common sterols in plants that often exist in a mixture [29,30]. The only difference between these two compounds is that stigmasterol has an extra double bond at C-22 (δ_C_ 138.46) and C-23 (δ_C_ 129.42) over β-sitosterol. Studies have also shown that obtaining β-sitosterol or stigmasterol as a pure form is difficult [31]. Compound **6** isolated from the hexane fraction was characterized as 1-octadecanol based on the analysis of NMR (1D/2D) and GC-MS data. It has been previously isolated from *Cassia sophera* heartwood extract [32] and identified by gas chromatography (GC) techniques from various plants [33,34,35].

**Figure 1:**
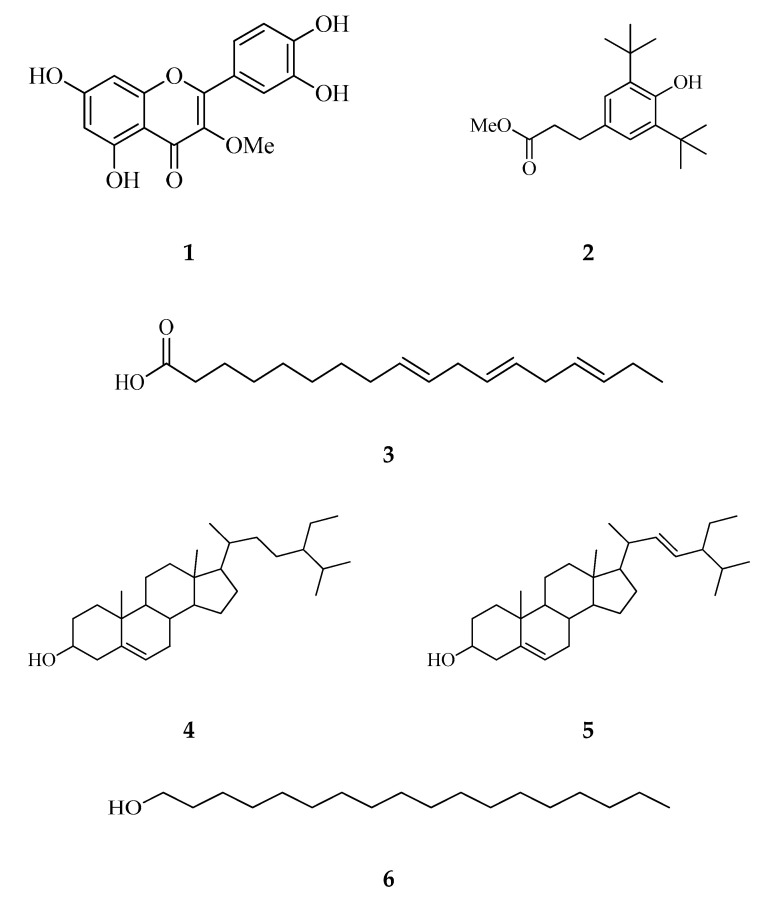
The structures of the isolated compounds; **1**–**6** of *C. timorensis* leaves and flowers.

**Figure 2:**
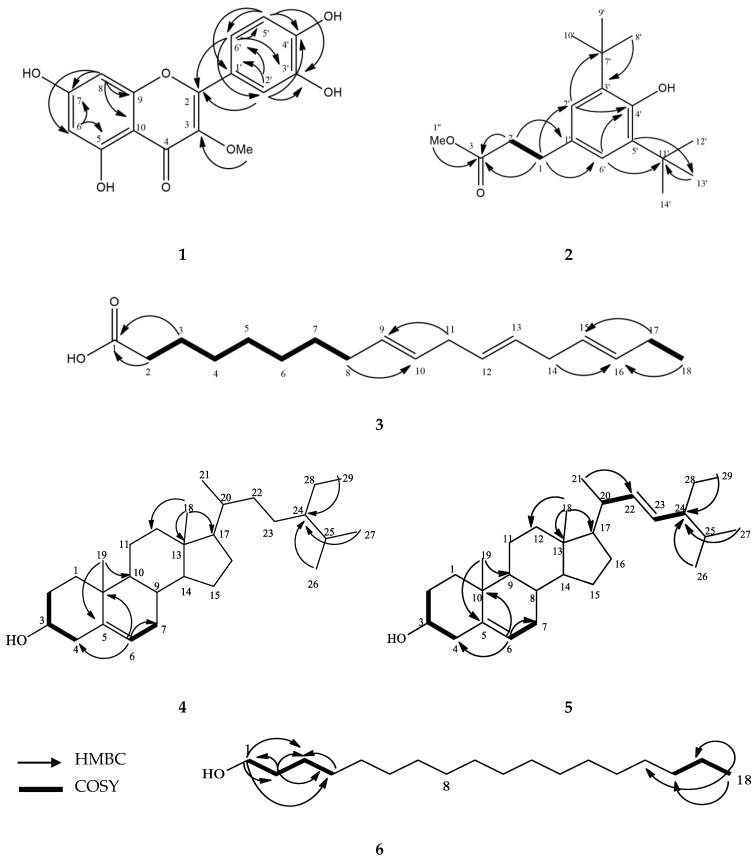
HMBC and COSY key correlations of compounds **1**–**6**.

To the best of our knowledge, this is the first report on the isolation of compounds **1**–**6** (Figure 1) from *C. timorensis*. The structure elucidation of the isolated compounds was done based on the UV, NMR (1D and 2D), and MS data as well as by comparison to the published data (Supplementary data). The key correlations of COSY and HMBC of compounds **1**–**6** can be found in Figure 2.

### 2.3. In Vitro Acetylcholinesterase Enzyme Inhibition Activity

The in vitro AChE inhibitory activity of the isolated compounds was evaluated at 0.1 mg/mL and their IC_50_ values were also determined (Table 3). Compound **1** demonstrated AChE inhibition with an IC_50_ of 83.71 µM. However, the activity of both **1** and quercetin are quite low compared to the standard drug galantamine (4.63 ± 0.03 µM). Compounds **2**, **3,** and **6** were found to have weak inhibitory activity against AChE, while **4** and **5** demonstrated moderate activity against AChE with 49.1% inhibition. Previous in vitro studies reported almost identical AChE inhibitory activities for compounds **4** and **5**, individually, where the IC_50_ of compound **4** was recorded at 55 µg/mL, while the IC_50_ for compound **5** was 63 µg/mL [36]. Despite their individual notable inhibiting activities against AChE reported in the literature, we failed to separate them. The IC_50_ values of compounds **2**–**6** could not be determined due to their low percentage inhibition activity, which was less than 50% inhibition at 0.1 mg/mL.

AChE inhibition of **1** was determined and compared with quercetin (of the same class of compounds). The activity of 3-methoxyquercetin is approximately three times higher than quercetin. The major difference between these two compounds based on their structure is only the substitution of the hydroxyl group at the C-3 position in quercetin with a methoxy group (–OCH_3_) in **1**. A major debate involving neurological disorders is the ability of the drug candidates to penetrate the blood-brain barrier (BBB). Faria and colleagues conducted an in vivo study on a rat endothelial RBE-4 cells BBB model and found that quercetin had the least efficiency in penetrating the BBB in a time-dependent manner compared to catechin and anthrocyanin due to its lower lipophilicity [37]. Permeability of a compound into the BBB is dependent on its lipophilicity. The less polar compounds, for example, the methylated flavonoids, are more lipophilic and have higher BBB permeability as compared to the more polar flavonoids. A recent study showed that sudachitin, aceronin, and nevadensin have high BBB permeability due to the substitution of a methyl group at both ring-A and ring-C on the 2-phenyl chroman structure, which increases the lipophilicity of the compounds [38]. In another study, the presence of a hydroxyl group (–OH) at the C-3 position did not give any impact on the inhibition of AChE [39]. In the present study, the methoxy group substitution at the C-3 position in **1** did increase the inhibition of AChE as compared to the more polar quercetin.

The evaluation of flavonoids as acetylcholinesterase inhibitors (AChEIs) has been widely investigated. In 2011, Uriarte-Pueyo and Calvo reported the ability of 128 flavonoid compounds, including those of subgroup classes such as flavones, flavanones, flavonols, isoflavones, and chalcones as AChEIs. They found that the inhibitory activity of flavones is higher than the flavanones that have a double bond at C-2–C-3 of the C-ring position, which is important in impeding AChE activity [40]. The activity of flavonoids as AChE inhibitors were also reported by Balkis and co-workers [41]. They concluded that the activity of the compounds depends on four main aspects: (1) the presence of an unsaturated 2-phenyl chroman structure; (2) the presence of a hydroxyl group attached at C-5, C-6, and C-7 positions on the A-ring; (3) the insignificance of the hydroxyl group present in the B-ring, and (4) the presence of a substituted group at the C-3 position of the unsaturated C-ring [41]. **1** showed the characteristic of an unsaturated 2-phenyl chroman structure with the attachment of a hydroxyl group at C-5 and C-7. Importantly, **1** has a substitution of a methoxy group at the C-3 position, which possibly contributes to the inhibitory activity against AChE.

### 2.4. In Silico Molecular Docking

The active site of the protein AChE is characterized by a deep and narrow gorge, approximately 20 Å, that penetrates half of the AChE and widens out to the base [42]. The entry gate of the active site is known as the peripheral anionic site (PAS) and consists of several important amino acids including TYR70, ASP72, TYR121, and TRP279 [43]. In addition, AChE is made up of two subsites; anionic and esteratic subsites that correspond to the choline-binding and catalytic pockets, respectively. The anionic subsite consists of an important amino acid, TRP84, which is highly conserved. TRP84 is one of the most significant amino acids in the AChE binding pocket for the hydrophobic cationic-π interaction to occur. Meanwhile, the esteratic subsite has two binding pockets; the oxyanion hole (GLY116, GLY117 and ALA199) and the acyl pocket (PHE330 and PHE 331), which are responsible for the stabilization of the substrate so that hydrolyzation can take place. The esteractic subsite is also responsible for the hydrolysis of the ACh neurotransmitter at its catalytic triad (SER200-HIS440-GLU327) [44].

**Figure 3:**
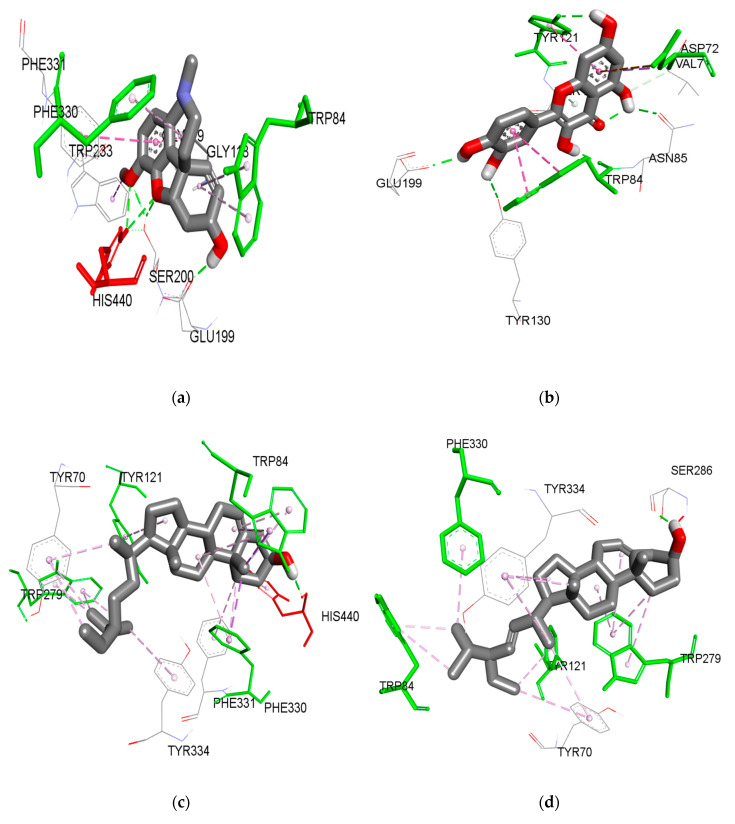
Interactions of compound (**a**) galanthamine, (**b**) 3-methoxy quercetin, (**c**) β-sitosterol, and (**d**) stigmasterol with PAS (amino acid in green) and the acyl pocket (amino acid in red) in the active site of TcAChE. The dotted green line is the hydrogen bond, purple is the Pi-sigma bond, and pink is the Pi-alkyl bond interaction.

Docking analysis of galantamine, **1**, and quercetin are presented in Figure 3. Compound **1** bound to the AChE pocket with the lowest free energy of binding (FEB) of −8.43 kcal/mol, similarly to galantamine (−9.63 kcal/mol). The 3D visualization analysis showed that **1** formed an interaction with the PAS, which includes several important amino acids; ASP72, TYR121 and PHE288. TYR121 formed a hydrogen bond with the methoxy group located at the C-3 position of the C-ring with a distance of 2.46 Å. A hydrogen bond was also formed between the hydrogen atom of the 7-OH group of the A-ring with the ASP72 at a distance of 1.75 Å. Another hydrogen bond was formed between the oxygen atom 3′-OH of the B-ring with the acyl pocket (PHE288) of the active site gorge with a distance of 2.78 Å. These three hydrogen bonds might explain the inhibitory activity of compound **1** as observed from the in vitro assay. In contrast, quercetin (−8.38 kcal/mol) was found to form two hydrogen bonds between 7-OH and TYR121 (PAS), and also hydrophobic interaction with ASP72 (PAS). Other interactions were observed from 3′-OH of the B-ring with TYR130 and a hydrophobic interaction with TRP84 at the choline binding site.

For comparison, galantamine bound to the active site pockets with the lowest FEB at −9.63 kcal/mol. A strong hydrogen bond between the hydroxyl oxygen of galantamine to the GLU199 was observed with a distance of 1.77 Å. Apart from that, the oxygen group from the methoxy group of galantamine formed a hydrogen bond with the anionic subsite amino acid (TYR130). The double bond of cyclohexene (C1=C2) of the galantamine faced toward the indole ring of TRP84 and stacked against the pi system of the TRP84, forming a favorable hydrophobic interaction. In addition, the oxygen atom from the methyl group and the oxygen atom of the tetrahydrofuran group of galantamine are involved in hydrogen bonding with both the catalytic residues (SER200 and HIS440). In addition, galantamine also aligned in a planar position and was stabilized via a hydrophobic interaction with the oxyanion hole and acyl pocket residues (GLY118, PHE330, and PHE331).

Based on the docking results, **1** was bound to PAS and sandwiched between the acyl pocket of PHE288 halfway along the active site gorge. Based on a recent finding by Neto and his colleagues, binding that occurs at PAS and the acyl pocket would obstruct the hydrolysis of neurotransmitters by blocking their pathway to the catalytic triad site located at the bottom of the active site [45]. Thus, the binding of **1** at this position will likely help to block the ACh neurotransmitters from entering the active site, hence prolonging their activity and ultimately improving the symptoms of AD [44]. In contrast, the interactions of quercetin were found mainly between the B-ring of quercetin and the choline binding site (TYR130 and TRP84). However, as discussed earlier, any interaction that occurs between the hydroxyl group of the B-ring with AChE active sites is not able to fully inhibit AChE [41].

On the other hand, galantamine primarily targets the anionic subsite and the esteratic subsite; (i) oxyanion hole, (ii) acyl pocket, (iii) catalytic triad. As observed, the flavonoid compounds, **1** and quercetin exert an inhibitory mechanism mainly at the PAS, acyl pocket, and choline binding site without any interaction with residues that make up the oxyanion hole and the catalytic triad. These docking results of both compounds and galantamine perhaps clarify the differences in the inhibitory activity exhibited by these three compounds in our in vitro results.

## 3. Materials and Methods

### 3.1. Materials (Chemicals) and Instruments

Acetylcholinesterase (AChE) from *Electrophorus electricus* (electrical eels) type VI-S (510 units/mg), substrate acetylthiocholine iodide (ATCI), phosphate buffer, sodium phosphate monobasic and sodium phosphate dibasic, quercetin, and galantamine were purchased from Sigma-Aldrich (St. Louis, MO, USA). Coloring agent 5-5′dithiobis [2-nitrobenzoicacid] (DTNB) was purchased from ACROS (Geel, Belgium). Silica gel (Kieselgel 60, 230–400 mesh atm) was purchased from Merck (Darmstadt, Germany). The solvents were of analytical grade and used as received.

NMR data were collected using either a Bruker Biospin spectrometer at 500 MHz for ^1^H and 125 MHz for ^13^C or Bruker advance III HD 700 MHz NMR spectrometer, equipped with a 5-mm BBO probe, operating at 700 MHz for ^1^H and 175 MHz for ^13^C. Residual solvent signals were applied for referencing. MS data were acquired using a UPLC-H-Class system (Waters, Millford, MA, USA) on a C18 column (i.d 2.1 mm × 50 mm, 1.7 µm particle sizes). The elution was done using a linear gradient system (acetonitrile; 0.05% aqueous formic acid: 5 to 100% for 4 min at 0.5 mL/min). The injected analytes absorbed UV and were detected by a UV/Vis detector, proportional to the peak signals produced on the chromatogram. The analytes were then converted and fragmented into charged ions by the ESI interface and were brought into the mass spectrometer (Triple Quad™ LC-MS/MS system). A positive ion mode mass spectrum was generated. MS data were also obtained using GC (HP 6890 series GC system, Hewlett-Packard, Palo Alto, CA, USA), equipped with an autosampler (HP 7683 series injector) and coupled with a mass selective detector (HP 5973) using a cross-linked 5% phenylmethylsiloxane capillary column (30 m × 0.25 mm i.d., 0.25 film thickness).

### 3.2. Plant Materials

Leaves, stems, fruits, and flowers of *C. alata* L. Roxb were collected from Pahang, while *C. timorensis* (DC.) H. S Irwin & Barneby, *C. spectabilis* (DC.) H. S Irwin & Barneby, *C. grandis* L. f., and *C. fistula* L. were collected from Universiti Sains Malaysia (USM), and identified by a USM botanist, Mr. Shunmugan Vellosamy, from the Herbarium Department of the School of Biological Sciences, USM. The leaves, stems, and flowers were air-dried for 7 days, while the fruits were freeze-dried for 72 h. Dried samples were powdered to increase surface area during the extraction process.

### 3.3. Extraction of Cassia Species for Screening

Two hundred grams of each plant sample was soaked in 99.9% methanol for 3 days and filtered using Whatman filter paper no 1. The extraction process was performed three times and the filtrates were pooled together. Then methanol was evaporated under pressure at 40 °C to obtain a crude methanolic extract. The crude extracts were stored at 5 °C prior to use.

### 3.4. Collection, Extraction and Fractionation of C. timorensis

*C. timorensis* leaves and flowers collected from Universiti Sains Malaysia were identified and approved as *C. timorensis*. The voucher specimen (No: 11778) was deposited in the Herbarium, School of Biological Sciences, Universiti Sains Malaysia. Briefly, air-dried leaves (1 kg) and flowers (300 g) of *C. timorensis* were extracted by soaking in methanol at room temperature for three days followed by filtration using Whatman filter paper no. 1 to separate the plant materials. This process was repeated thrice. Then, the filtrates were combined and dried under reduced pressure at 40 °C to yield methanolic extracts (100 g for leaves and 30 g for flowers). The crude extracts were stored at 4 °C until further use. The methanol extracts of *C. timorensis* (100 g for leaves and 30 g for flowers) were reconstituted in water:methanol (9:1) followed by liquid–liquid partitioning with *n*-hexane, ethyl acetate and butanol. Each fractionation was conducted three times with 250 mL of the respected solvent. The fractions from the same solvent were brought together and dried under vacuum before being screened for their anti-cholinesterase activity.

### 3.5. Isolation from the Ethyl Acetate Fraction of C. timorensis Leaves

The ethyl acetate fraction (14 g) was purified by using column chromatography (silica gel 60, 230–400 mesh ASTM). The elution was done with a stepwise gradient solvent system using chloroform:methanol, providing five major fractions; fraction 1 (100% chloroform), fraction 2 (90% chloroform), fraction 3 (85% chloroform), fraction 4 (80% chloroform) and fraction 5 (50% chloroform). Fraction 4 was further purified using column chromatography (silica gel 60, 230–400 mesh ASTM) and eluted with a gradient solvent system of *n*-hexane:ethyl acetate (100% hexane up to 100% ethyl acetate *v*/*v*) to give five major sub-fractions. Purification of sub-fraction 2 yielded 3-methoxyquercetin (21.4 mg) (**1**).

### 3.6. Isolation from the n-Hexane Fraction of C. timorensis Leaves

The *n*-hexane fraction (0.844 g) was dissolved in hexane:acetone (1:1) and packed with silica gel into a Redisep^®^ solid load cartridge. The sample was further separated using medium pressure liquid chromatography (MPLC) (CombiFlash Rf, Teledyne ISCO). The elution was performed with hexane (A): acetone (B) as the mobile phase in a stepwise gradient method (0–100% solvent B for 30 min) using a 24 g high performance gold silica column (RediSep^®^ Rf, Teledyne ISCO). Five major fractions were collected. Fraction 2 and 4 were then further purified by using MPLC (13 g C18 column, Redisep^®^ Rf column) with a solvent system of acetonitrile:water (0.05% formic acid). Sub-fraction 1 from fraction 2 yielded benzenepropanoic acid (2.2 mg), (**2**) whilst sub-fraction 7 from fraction 4 yielded octadecatrienoic acid (8.3 mg) (**3**).

### 3.7. Isolation of Compounds of C. timorensis Flowers

The ethyl acetate fraction of *C. timorensis* flowers (6.00 g) was subjected to a silica gel 60 (mesh 230–400 ASTM) column and eluted with a mixture of solvents of increasing polarity of *n*-hexane:ethyl acetate (100% hexane up to 100% ethyl acetate (*v*/*v*)). The eluted fractions were monitored on TLC (silica gel 60 F_254_, Merck) using *n*-hexane:ethyl acetate. Fractions with similar TLC profiles were combined and pooled together to give 30 sub-fractions. Sub-fraction 9 was purified by crystallization, yielding white needle crystals. Based on spectral data analysis as well as by comparison to literature data, the crystals of sub-fraction 9 were identified as a mixture of β-sitosterol (12 mg) (**4**) and stigmasterol (12 mg) (**5**). Moreover, the *n*-hexane fraction of *C. timorensis* flowers (3.00 g) was subjected to column chromatography (silica gel 60, 230–400 ASTM) to yield 25 sub-fractions. The fractions were eluted using a gradient mobile phase starting from 100% *n*-hexane until 100% ethyl acetate was reached. Purification and crystallization of sub-fraction 8 gave compound **6**, which was identified as 1-octadecanol (10 mg). On the other hand, purification and crystallization of sub-fraction 13 again gave the mixture of sterol (compounds **4** and **5**), which was isolated before from the ethyl acetate fraction.

### 3.8. In Vitro Acetylcholinesterase Assay

The in vitro AChE inhibition assay was evaluated using a spectrophotometry method developed by Ellman with some modifications [41]. The assay was conducted in a 96-well plate with a total volume of 200 µL assay mixtures in comparison to galantamine as the positive control. Plant extracts were dissolved in dimethyl sulfoxide (DMSO) and tested at a final concentration of 0.2 mg/mL, while compounds were tested at 0.1 mg/mL. The final concentration of DMSO was fixed at 1% in the 96-well plate. In a 96-well plate, 178 µL of 50 mM phosphate buffer, 2 µL of 1 mg/mL compound and 20 mg/mL extract, and 10 µL of 0.5 U/mL AChE were added. The first incubation period was 15 min at 25 °C. Five microliters of 14 mM ATCI substrate and 10 mM of the color indicator, DTNB, were added equally into each well and the well was then incubated at 25 °C for 30 min to initiate the enzyme reaction. The absorbance was measured at 415 nm using Promega Glomax^®^ Multi Plus Reader (Promega, Madison, WI, USA). Each sample was done in triplicate and eight 2-fold serial dilutions were performed for each sample to determine the fifty-percentage inhibitory concentration (IC_50_). The percentage of inhibition was calculated using formula (1) [42]:

(1) $$\mathrm{Percentage}\;\mathrm{Inhibition}\;(\%)\;=\;\frac{Absorbance\;of\;negative\;control\;-\;Absorbance\;of\;test\;sample}{\;Absorbance\;of\;negative\;control}\;\times \;100$$

### 3.9. Statistical Analysis

IC_50_ values of plant crude extract, fractions, and compounds were calculated from the graph of percentage of inhibition versus extract concentrations using Microsoft Excel. GraphPad Prism software was used to generate the graphs. The IC_50_ values and the mean ± s.d. were calculated at 95% confidence interval.

### 3.10. In Silico Molecular Docking

The 3D structure of *Torpedo californica* acetylcholinesterase (TcAChE) in complex with galanthamine derivatives (PDB: 1W6R; 2.05 Å, [46]) was retrieved from Research Collaboratory for Structural Bioinformatics (RCSB) Protein Data Bank (http://www.rcsb.org/). The galantamine derivative bound in the crystal structure was taken out from the complex and saved as a PDB file using Discovery Studio 2.5 and assigned with Gasteiger charges using AutoDockTools 1.5.6 and later redocked to the protein as a control docking using AutoDock4.2 [47]. Other ligands and water molecules present in the protein complex were also removed. Polar hydrogen atoms and Kollman charges were then added to the protein. The grid box size was set to 90 × 90 × 90, with a grid spacing of 0.375 Å, and the center of mass of the binding pocket was set to 3.518, 65.122, 64.481 (x, y, z, respectively). The docking parameter was set to default, allowing 250 conformations by using the genetic algorithm search parameter. The 2D structure of galantamine, quercetin and **1**, were acquired from the PubChem database (https://pubchem.ncbi.nlm.nih.gov/) and converted to 3D using Discovery Studio 3.5, which was also used to visualize the 2D and 3D interactions for the lowest binding energy of the selected compounds with the binding pocket of the TcAChE.

## 4. Conclusions

Screening of *C. timorensis* for acetylcholinesterase inhibition in this study led to the identification of six compounds. As far as we are aware, all of these compounds were newly isolated from the plant since the first documented isolation of *C. timorensis* in 1984. The in vitro AChEI assay showed that compounds **2**, **3**, and **6** are not active, whilst **4** and **5** showed moderate activity as a mixture. Compound **1** was found to be active with an IC_50_ of 83.71 µM. The ability of **1** to inhibit AChE in vitro was then simulated in silico against the targeted enzyme (TcAChE) to understand the inhibitory mechanism at the molecular level. Compound **1** displayed protein-ligand interactions at PAS and with the acyl pocket of the active sites. The interaction at these positions will likely obstruct the hydrolysis of the ACh neurotransmitter as it blocks the entrance and the exit from the enzyme active sites.

## Figures and Tables

**Table 1:** Percentage inhibition of methanol extracts of *Cassia* spp. at 0.2 mg/mL.

Plants	Part	Percentage Inhibition (%) ^1^	Strength of Inhibition
*C. timorensis*	Leaves	94.69 ± 3.08	Good
*C. timorensis*	Stems	97.16 ± 2.08	Good
*C. timorensis*	Flowers	96.94 ± 0.73	Good
*C. timorensis*	Fruits	49.09 ± 1.64	Moderate
*C. grandis*	Leaves	86.09 ± 0.66	Good
*C. grandis*	Stems	91.76 ± 0.73	Good
*C. fistula*	Leaves	20.63 ± 6.68	Poor
*C. fistula*	Stems	64.32 ± 0.09	Good
*C. fistula*	Flowers	8.72 ± 1.10	Poor
*C. fistula*	Fruits	19.48 ± 2.86	Poor
*C. spectabilis*	Leaves	45.96 ± 8.20	Moderate
*C. spectabilis*	Stems	27.18 ± 4.79	Moderate
*C. spectabilis*	Fruits	37.32 ± 2.07	Moderate
*C. alata*	Leaves	26.40 ± 6.65	Moderate
*C. alata*	Stems	18.43 ± 7.42	Poor
*C. alata*	Flowers	29.01 ± 3.05	Moderate
*C. alata*	Fruits	27.86 ± 1.63	Moderate

^1^ Data reported as mean ± s.d. (*n* = 3).

**Table 2:** Percentage inhibitions of *C. timorensis* fractions at 0.2 mg/mL.

Plants	Fractions	Percentage Inhibition (%) ^1^
*C. timorensis* leaves	Hexane	30.92 ± 4.92
	Ethyl acetate	98.98 ± 0.57
	Butanol	91.83 ± 1.50
	Aqueous	98.80 ± 0.55
*C. timorensis* stems	Hexane	26.66 ± 1.70
	Ethyl acetate	30.84 ± 2.83
	Butanol	94.92 ± 1.60
	Aqueous	84.35 ± 1.26
*C. timorensis* flowers	Hexane	92.35 ± 0.01
	Ethyl acetate	95.98 ± 1.30
	Butanol	98.16 ± 0.05
	Aqueous	38.32 ± 0.09

^1^ Data reported as mean ± s.d. (*n* = 3).

**Table 3:** IC_50_ values of compound **1** against AChE compared to quercetin and galantamine.

Compound	AChE Inhibition (IC_50_) µM
1	83.71 ± 4.67
Quercetin	249.10 ± 27.14
Galantamine	4.63 ± 0.10

Data reported as mean ± s.d. (*n* = 3).

## Supplementary Materials

The following are available online. Scheme S1. Elution scheme for isolation of *C.timorensis* leaf and flowers extracts, Figure S1. Mass spectrum of 3-methoxy quercetin (**1**), Figure S2. ^1^H-NMR of 3-methoxyquercetin (**1**) [500 Hz, acetone-d6], Figure S3. ^13^C-NMR of 3-Methoxyquercetin (**1**) [125 MHz, acetone-d6], Figure S4. ^1^H→^13^C HSQC NMR spectrum of 3-Methoxyquercetin (**1**) [500 MHz, acetone-d6], Figure S5. ^1^H→^13^C HMBC NMR spectrum of 3-Methoxyquercetin (**1**) [500 MHz, acetone-d6], Figure S6. ^1^H→^1^H COSY NMR spectrum of 3-Methoxyquercetin (**1**) [500 MHz, acetone-d6], Figure S7. Mass spectrum of Benzenepropanoic acid (**2**), Figure S8. ^1^H-NMR of Benzenepropanoic acid (**2**) [500 MHz, CDCl3], Figure S9. ^13^C-NMR of Benzenepropanoic acid (**2**) [125 MHz, CDCl3], Figure S10. ^1^H→^13^C HSQC NMR spectrum of Benzenepropanoic acid (**2**) [500 MHz, CDCl3], Figure S11. ^1^H→^13^C HMBC NMR spectrum of Benzenepropanoic acid (**2**) [500 MHz, CDCl3], Figure S12. ^1^H→^1^H COSY NMR spectrum of Benzenepropanoic acid (**2**) [500 MHz, CDCl3], Figure S13. Mass spectrum of 9,12,15-Octadecatrienoic acid (**3**), Figure S14. ^1^H-NMR of 9,12,15-Octadecatrienoic acid (**3**) [500 MHz, CDCl3], Figure S15. ^13^C-NMR of 9,12,15-Octadecatrienoic acid (**3**) [125 MHz, CDCl3], Figure S16. ^1^H→^13^C HSQC NMR spectrum of 9,12,15-Octadecatrienoic acid (**3**) [500 MHz, CDCl3], Figure S17. ^1^H→^13^C HMBC NMR spectrum of 9,12,15-Octadecatrienoic acid (**3**) [500 MHz, CDCl3], Figure S18. ^1^H→^1^H COSY NMR spectrum of 9,12,15-Octadecatrienoic acid (**3**) [500 MHz, CDCl3], Figure S19. Mass spectrum of *β*-sitosterol (**4**), Figure S20. Mass spectrum of stigmasterol (**5**), Figure S21. ^1^H-NMR spectrum of the mixture of β-sitosterol (**4**) and stigmasterol (**5**) [700 MHz, CDCl_3_], Figure S22. ^13^C-NMR spectrum of the mixture of β-sitosterol (**4**) and stigmasterol (**5**) [175 MHz, CDCl_3_], Figure S23. ^1^H→^13^C HSQC NMR spectrum of β-sitosterol and stigmasterol [700 MHz, CDCl_3_], Figure S24. ^1^H→^13^C HMBC NMR spectrum of β-sitosterol (**4**) and stigmasterol (**5**) [700 MHz, CDCl_3_], Figure S25. ^1^H→^1^H COSY NMR spectrum of β-sitosterol (**4**) and stigmasterol (**5**) [700 MHz, CDCl_3_], Figure S26. Mass spectrum of 1-octadecanol (**6**), Figure S27. ^1^H-NMR of 1-octadecanol (**6**) [700 MHz, CDCl_3_], Figure S28. ^13^C-NMR spectrum of 1-octadecanol (**6**) [175 MHz, CDCl_3_], Figure S29. ^1^H→^13^C HSQC NMR spectrum of 1-octadecanol (**6**) [700 MHz, CDCl_3_], Figure S30. ^1^H→^13^C HMBC NMR spectrum of 1-octadecanol (**6**) [700 MHz, CDCl_3_**]**, Figure S31. ^1^H→^1^H COSY NMR spectrum of 1-octadecanol (**6**) [700 MHz, CDCl_3_].
