# Supplementary material for: Potential Anti-Acetylcholinesterase Activity of Cassia timorensis DC
Source: Molecules. 2020 Oct 4;25(19):4545. doi: 10.3390/molecules25194545 (PMC7582324; doi:10.3390/molecules25194545)
Supplement: Supplementary file 1 [file molecules-25-04545-s001.pdf]

Supplementary information

^{1}H-NMR and ^{13}C-NMR data of isolated compounds

3-Methoxyquercetin **(1):** yellow needles crystal; UV (MeOH) λ_{max} 255, 357 nm; MS (ESI) calculated for $C_{16}H_{12}O_7$ $[M + H]^-$ *m/z* 317.4; ^{1}H-NMR (500 MHz, C_3D_6O): δ 3.87 (s, 3H, OCH_3-3), 6.26 (d, 1H, *J*= 2.2, H-6), 6.50 (d, 1H, *J*=2.2, H-8), 7.01 (d, 1H *J*=8.6, H-5'), 7.71 (d, 1H, *J*=2.2, H-2'), 7.60 (d, d, 1H, *J*=2.2 & 8.6, H-6'), 12.82 (s, 1H, OH-5); ^{13}C-NMR (125 MHz, C_3D_6O): δc 59.27, 93.53, 98.48, 104.92, 115.34, 115.40, 121.17, 122.07, 138.35, 145.05, 148.28, 155.88, 156.92, 162.28, 164.10, 178.61 ppm.

Benzenepropanoic acid (**2**): yellowish oil; UV (MeOH) λ_{max} 208, 276 nm; MS (ESI) calculated for $C_{18}H_{28}O_3[M\text{-}H]^-$ *m/z* 291.3; ^{1}H NMR (500 MHz, $CDCl_3$): δ 1.41 (s, 18H), 2.58 (t, 2H, *J*=8), 2.85 (t, 2H, *J*=8), 3.63 (s, 2H), 3.67 (s, 3H, OMe), 5.04 (s, 1H, OH), 6.97 (s, 2H); ^{13}C-NMR (125 MHz, $CDCl_3$): δc 30.53, 31.23, 34.53, 36.56, 51.80, 70.79, 125.01 (2C), 131.32, 136.12, 152.34, 173.90, 178.25 ppm.

9,12,15-Octadecatrienoic acid (**3**): yellowish oil; UV (MeOH) λ_{max} 205, 252 nm; MS (ESI) calculated for $C_{18}H_{30}O_2$ $[M – H]^-$ *m/z* 277.5; ^{1}H-NMR (500 MHz, $CDCl_3$): δ 0.96 (t, 3H, J= 7.5), 1.30 (m, 8H), 1.61 (m, 2H), 2.03 (q, 4H, *J*=5), 2.07 (m, 2H), 2.33 (t, 2H, *J*= 7.5), 2.79 (t, 4H, *J*= 6.5), 5.34 (m, 6H); ^{13}C-NMR (125 MHz, $CDCl_3$): δc 14.48, 20.76, 24.88, 25.57, 25.84, 27.41, 29.24, 29.29, 29.35, 29.78, 34.17, 127.34, 127.96, 128.47, 128.51, 130.46, 132.18, 179.71 ppm.

β-sitosterol (**4**) : white powder; m/z 414; ^{1}H-NMR (700 MHz, $CDCl_3$): δH 5.35 (m,1H), 3.53 (m, 1H), 0.86-1.10 (5×3H); ^{13}C-NMR (175 MHz, $CDCl_3$): δc 140.90 (C-5), 138.46 (C-22), 129.24, 121.87, 71.97, 56.92, 56.20, 50.31, 45.99, 42.47, 42.46, 39.93, 37.41, 36.30, 34.10, 31.82 (2), 32.08, 29.31, 28.40, 26.14, 24.46, 23.22, 21.24, 19.56, 19.19, 19.13, 12.41, 12.02 ppm.

Stigmasterol (**5**) : white needles; m/z 412; ^{1}H-NMR (700 MHz, $CDCl_3$): δH 5.35 (m,1H), 5.01 (dd, *J* = 8.8, 15.2 Hz, 1H) and 5.13 (dd, *J* = 8.8, 15.2 Hz, 1H) 3.53 (m, 1H) , 0.86-1.10 (m, 5×3H); ^{13}C-NMR

(175 MHz, $CDCl_3$): δc 140.9, 138.46, 129.24, 121.87, 71.97, 56.80, 56.10, 51.39, 50.28, 42.46 (2), 40.46 , 39.83, 37.41 , 36.66 , 32.08 (2), 31.82 (2), 29.08, 24.52 , 25.57 , 21.24 , 21.37 (2), 19.98 , 18.94 , 12.12 , 12.14 ppm.

1-octadecanol (**6**) : white powder; m/z 269.90; ^{1}H-NMR (700 MHz, $CDCl_3$): δH 0.87(t, *J*=7 Hz, 3H), 1.25 (s, 30H), 1.56 (2H, m), 3.64 (t, *J*= 4.7 Hz, 2H); ^{13}C-NMR (175 MHz, $CDCl_3$) : δc 63.27, 32.96, 32.08, 29.52, 29.59, 29.76, 29.77, 29.82, 29.84, 29.86 (5), 25.88, 22.82, 14.28 ppm.

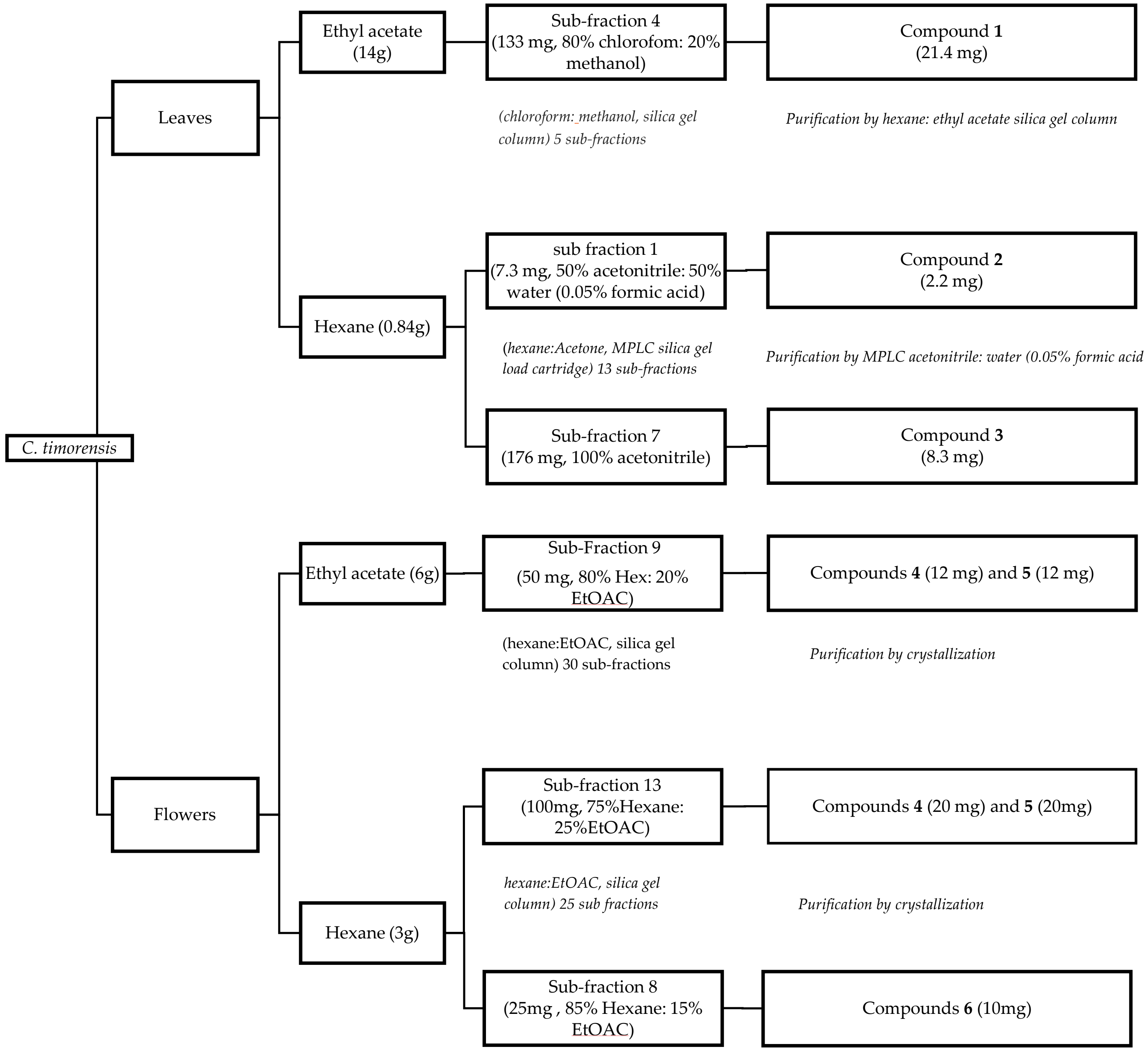

Scheme S1. Elution scheme for isolation of *C.timorensis* leaves and flowers extracts.

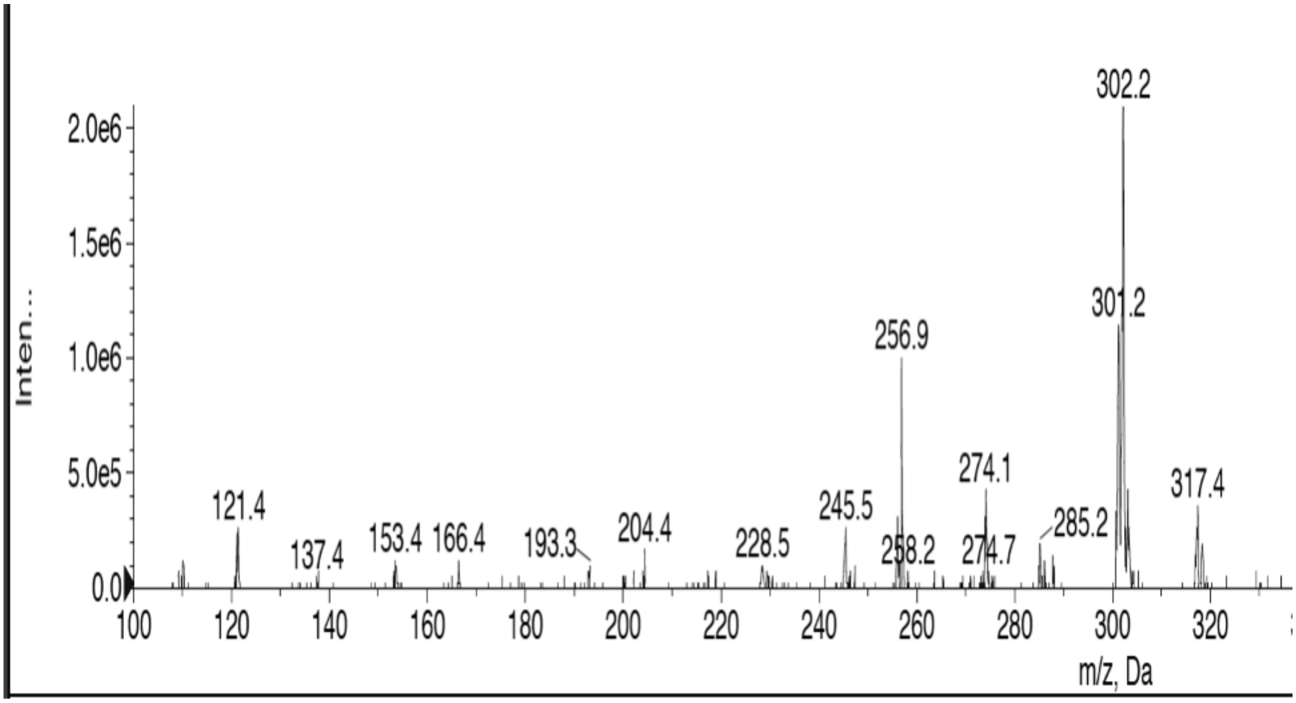

Figure S1. Mass spectrum of 3-methoxy quercetin **(1)**.

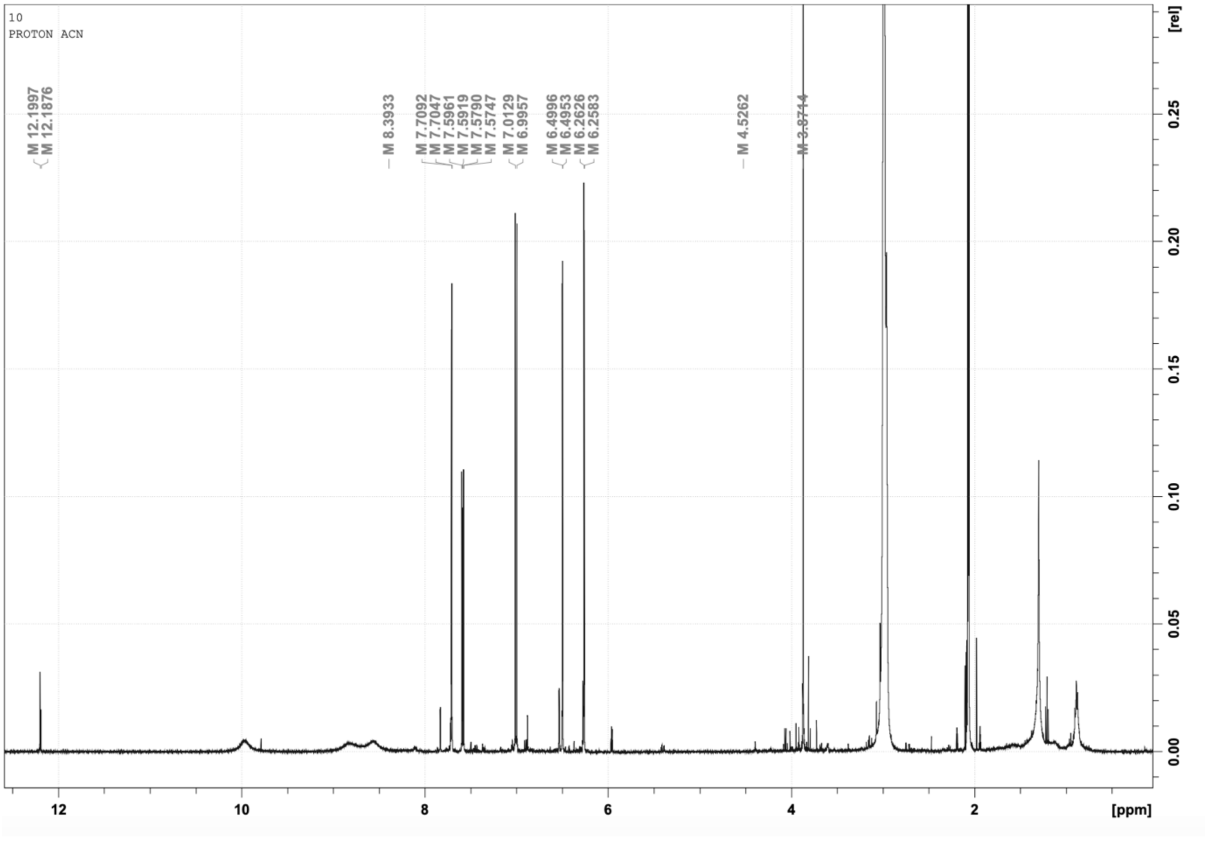

Figure S2. ^{1}H NMR of 3-Methoxyquercetin **(1)** [500 Hz, acetone-d6]

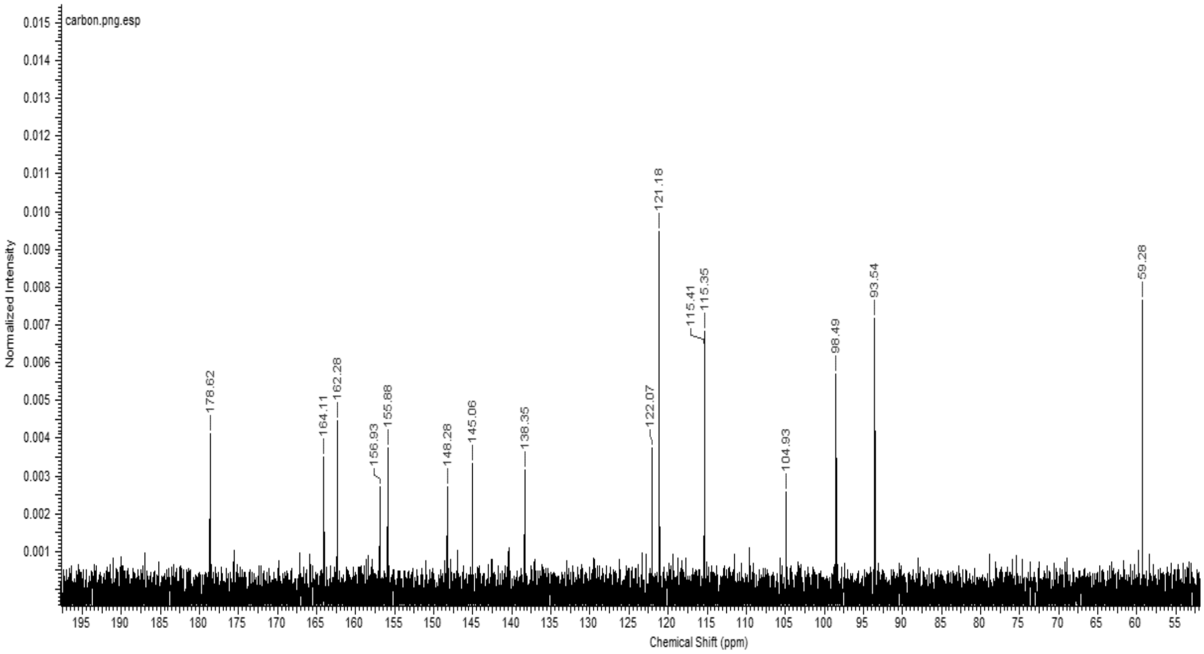

Figure S3. ^{13}C NMR of 3-Methoxyquercetin **(1)** [125 MHz, acetone-d6]

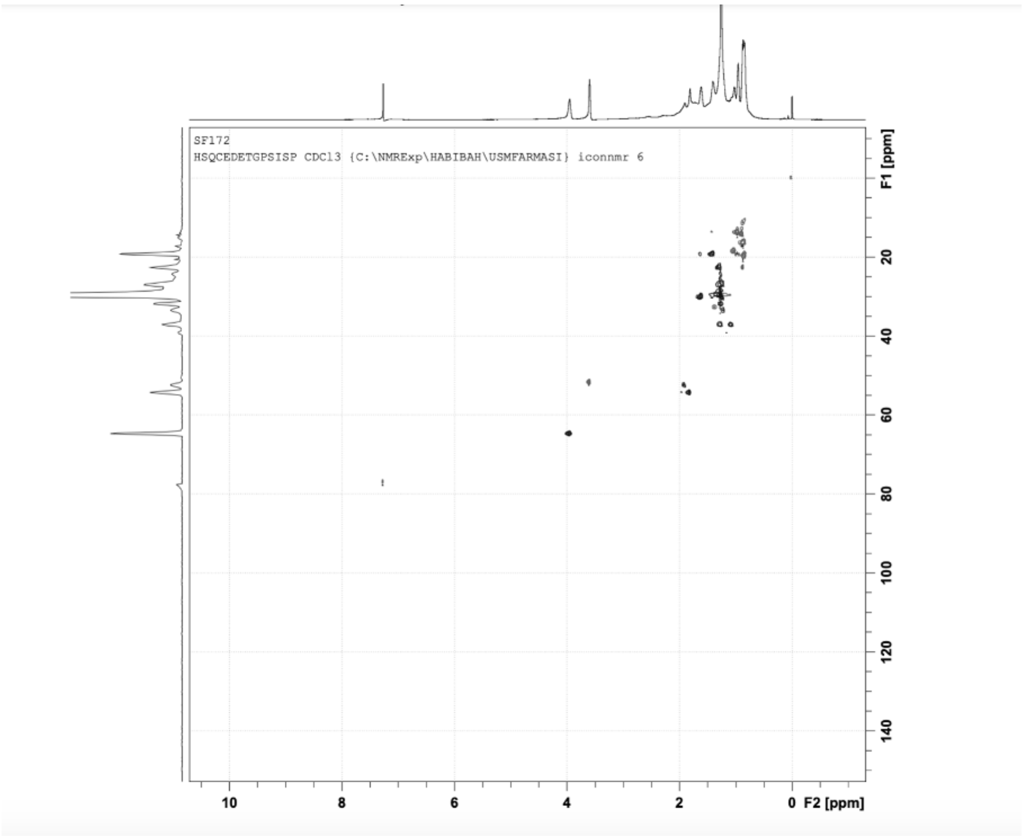

Figure S4. $^{1}H \rightarrow ^{13}C$ HSQC NMR spectrum of 3-Methoxyquercetin **(1)** [500 MHz, acetone-d6]

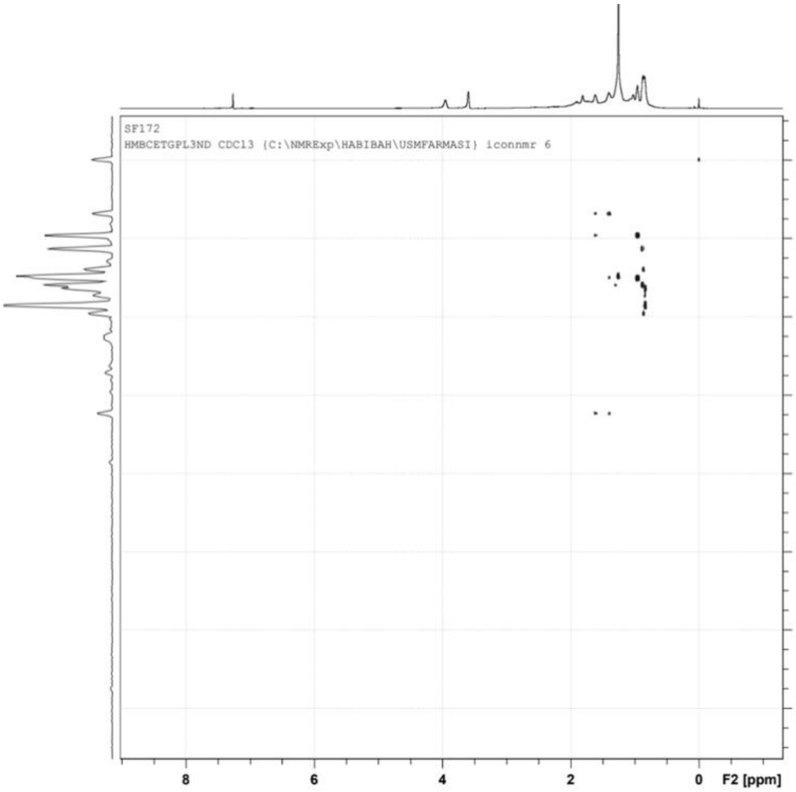

Figure S5. $^{1}H \rightarrow ^{13}C$ HMBC NMR spectrum of 3-Methoxyquercetin **(1)** [500 MHz, acetone-d6].

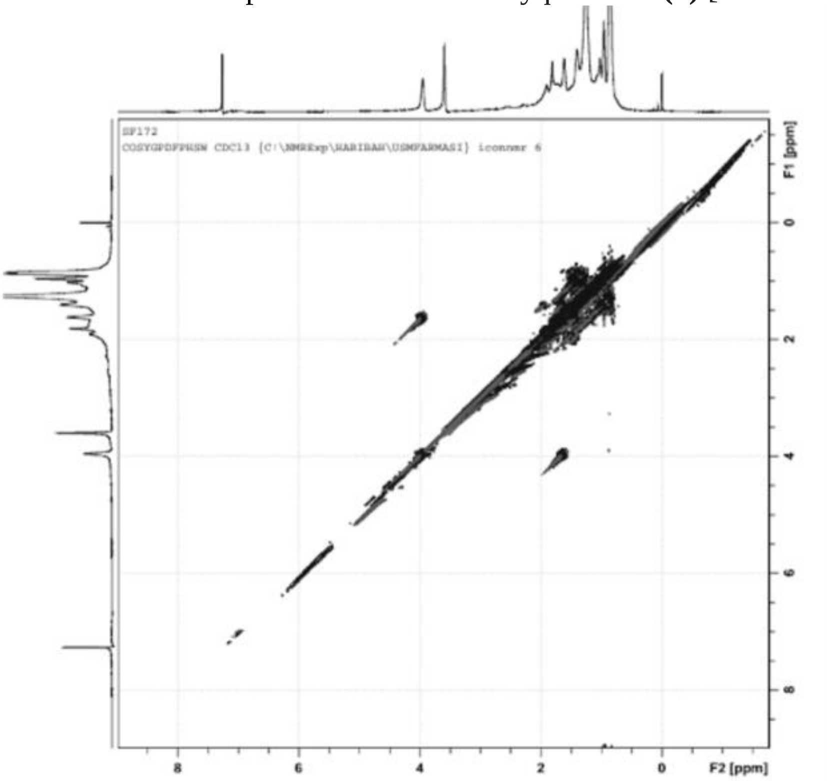

Figure S6. $^{1}H \rightarrow ^{1}H$ COSY NMR spectrum of 3-Methoxyquercetin **(1)** [500 MHz, acetone-d6].

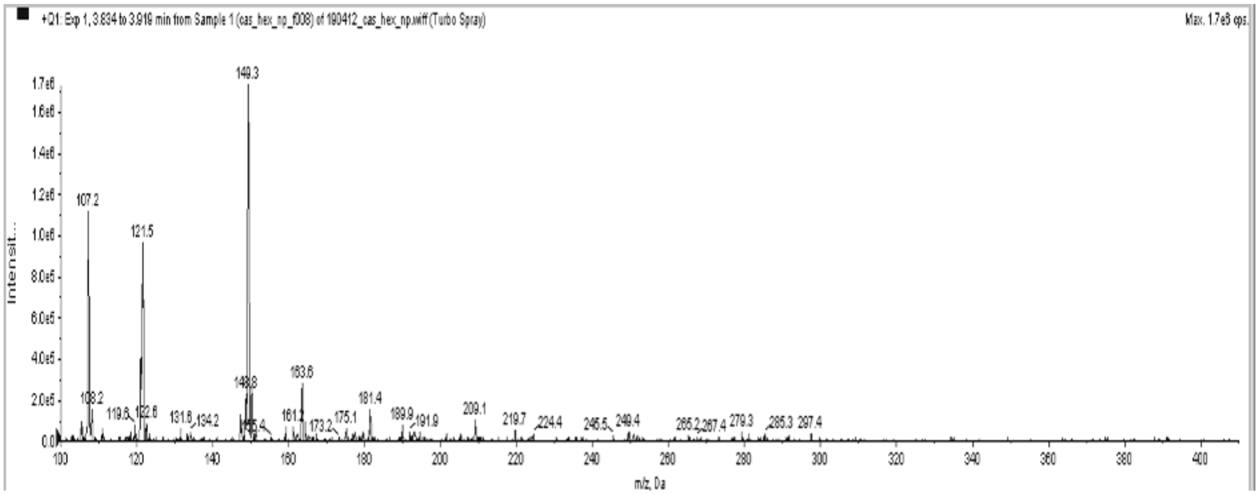

Figure S7. Mass spectrum of of Benzenepropanoic acid (**2**).

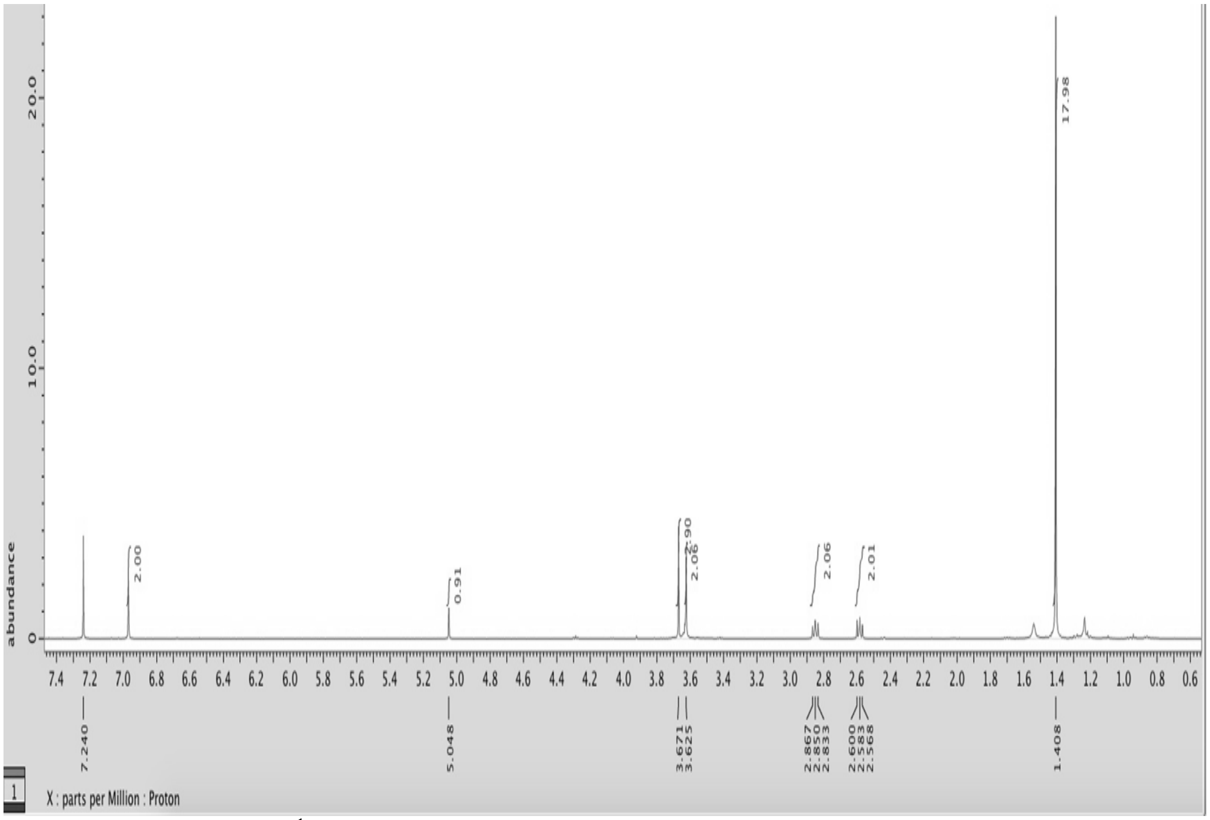

Figure S8. ^{1}H NMR of Benzenepropanoic acid (**2**) [500 MHz, CDCl3].

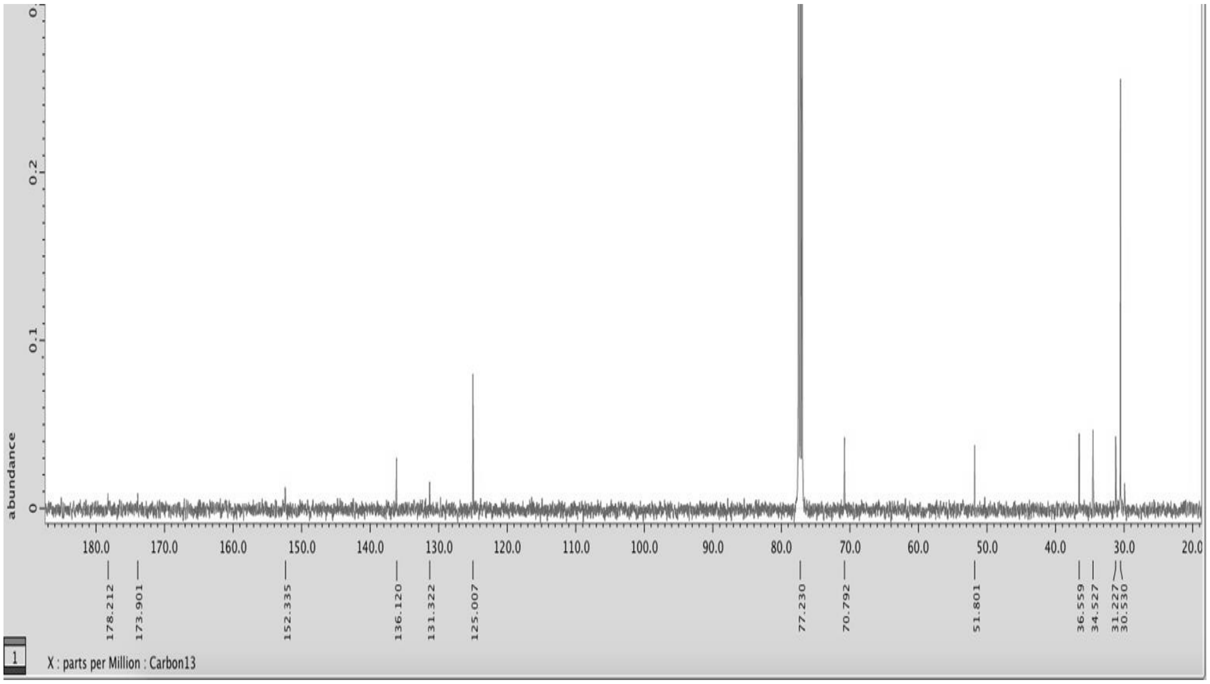

Figure S9. ^{13}C NMR of Benzenepropanoic acid (**2**) [125 MHz, CDCl3].

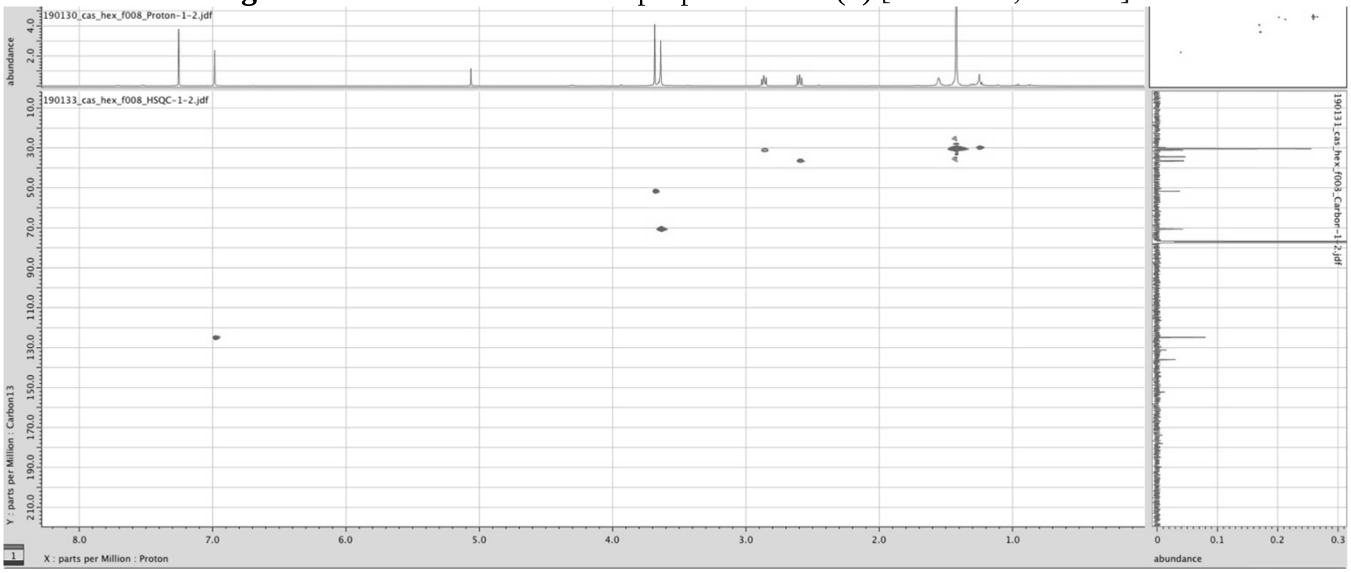

Figure S10. $^{1}H \rightarrow ^{13}C$ HSQC NMR spectrum of Benzenepropanoic acid (**2**) [500 MHz, CDCl3].

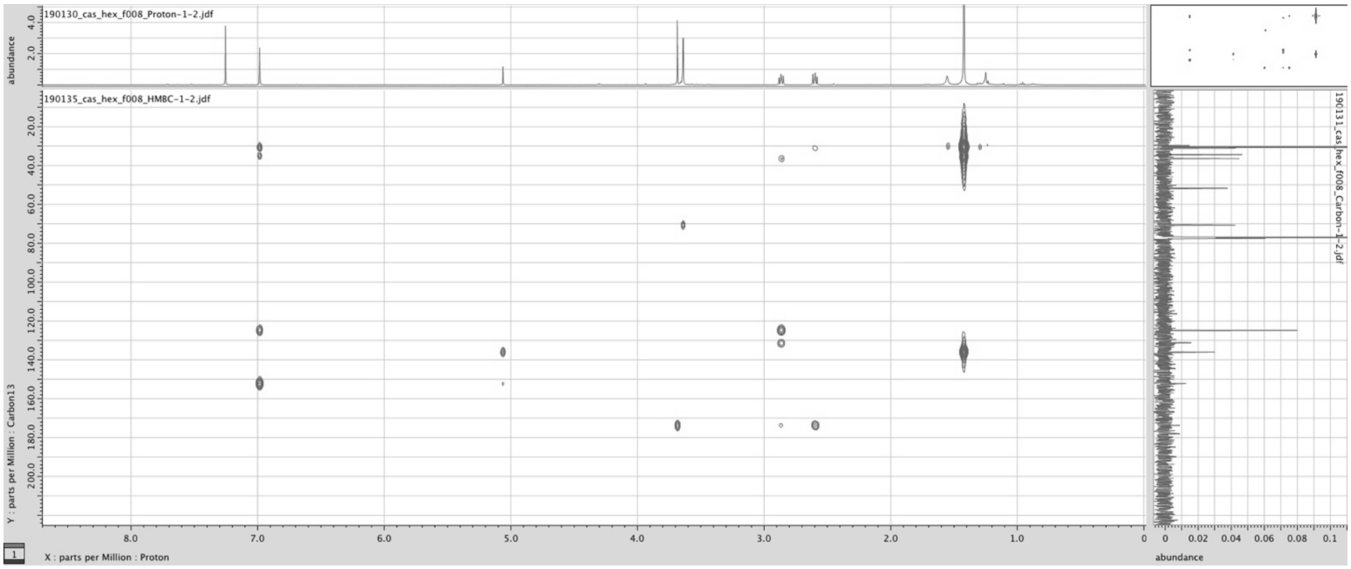

Figure S11. $^{1}H \rightarrow ^{13}C$ HMBC NMR spectrum of Benzenepropanoic acid (**2**) [500 MHz, CDCl3].

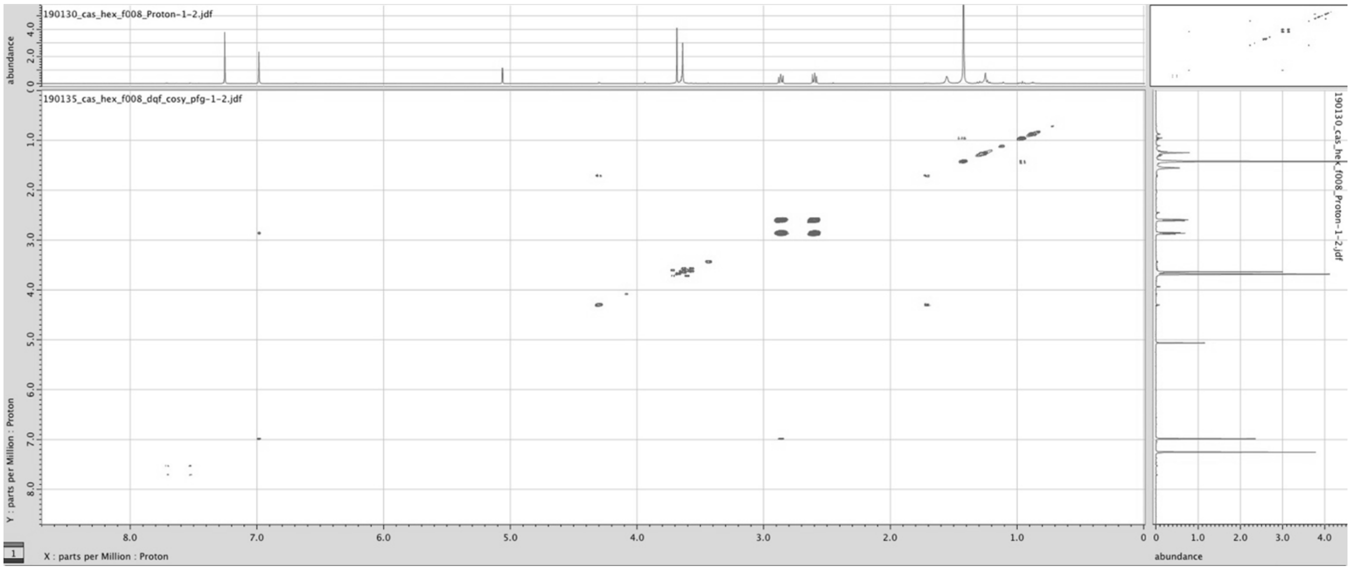

Figure S12. $^{1}H \rightarrow ^{1}H$ COSY NMR spectrum of Benzenepropanoic acid (**2**) [500 MHz, CDCl3].

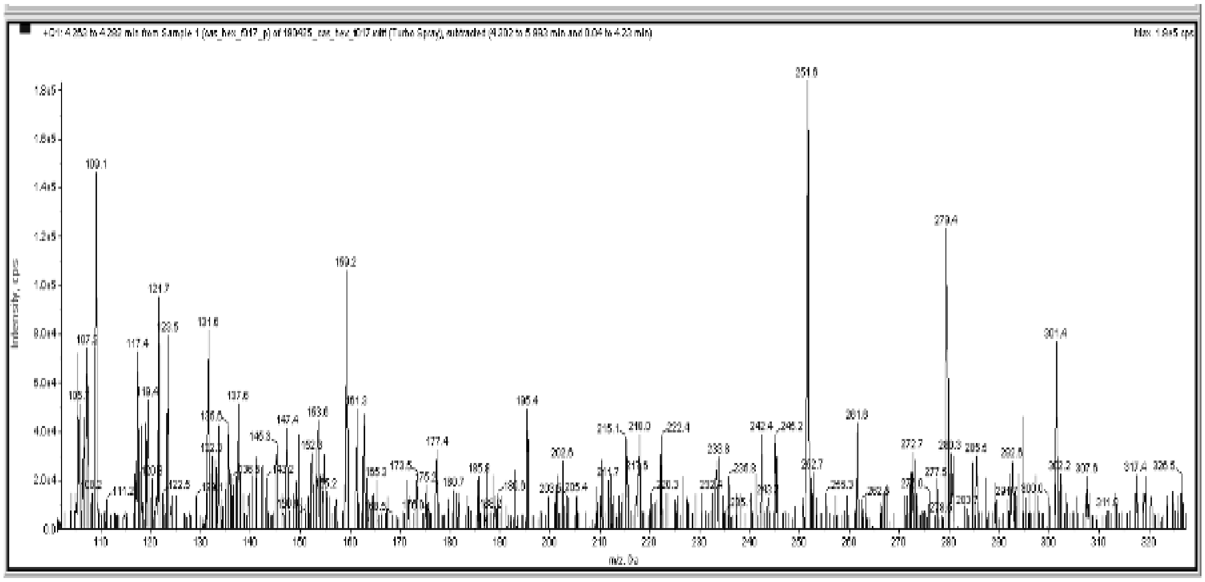

Figure S13. Mass spectrum of 9,12,15-Octadecatrienoic acid (**3**).

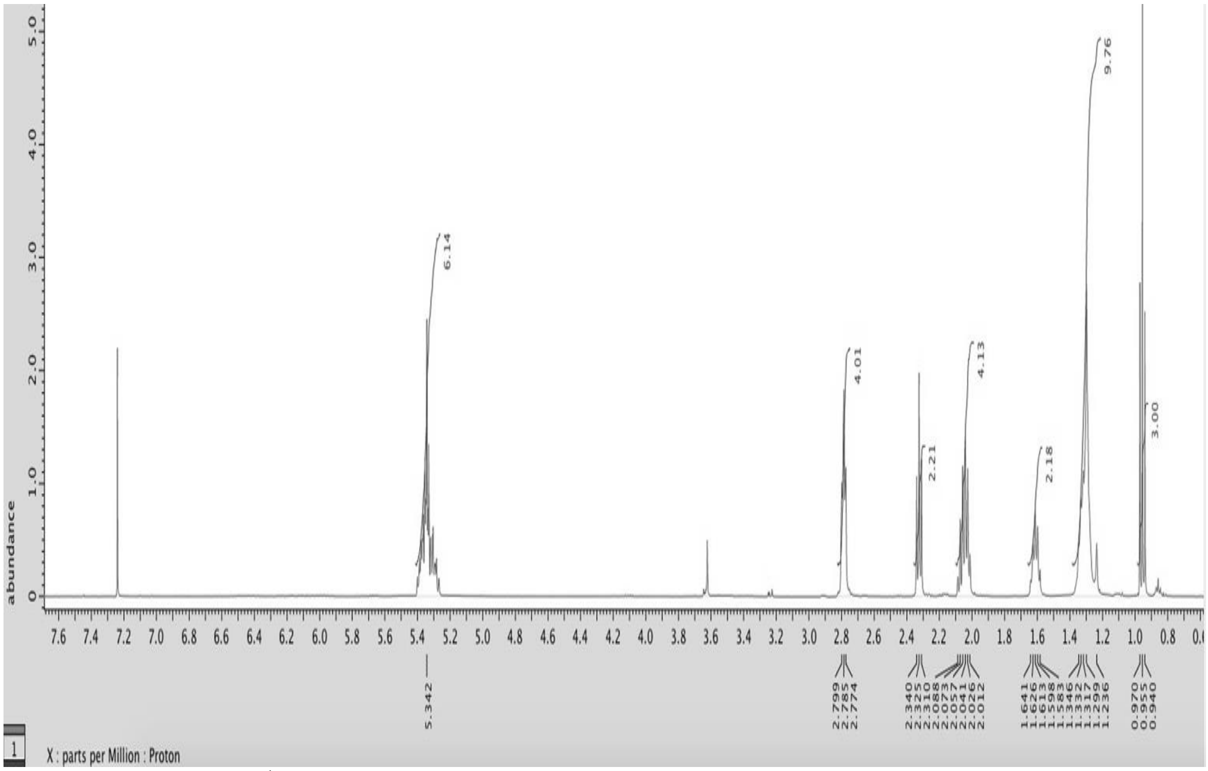

Figure S14. ^{1}H NMRof 9,12,15-Octadecatrienoic acid (**3**) [500 MHz, CDCl3].

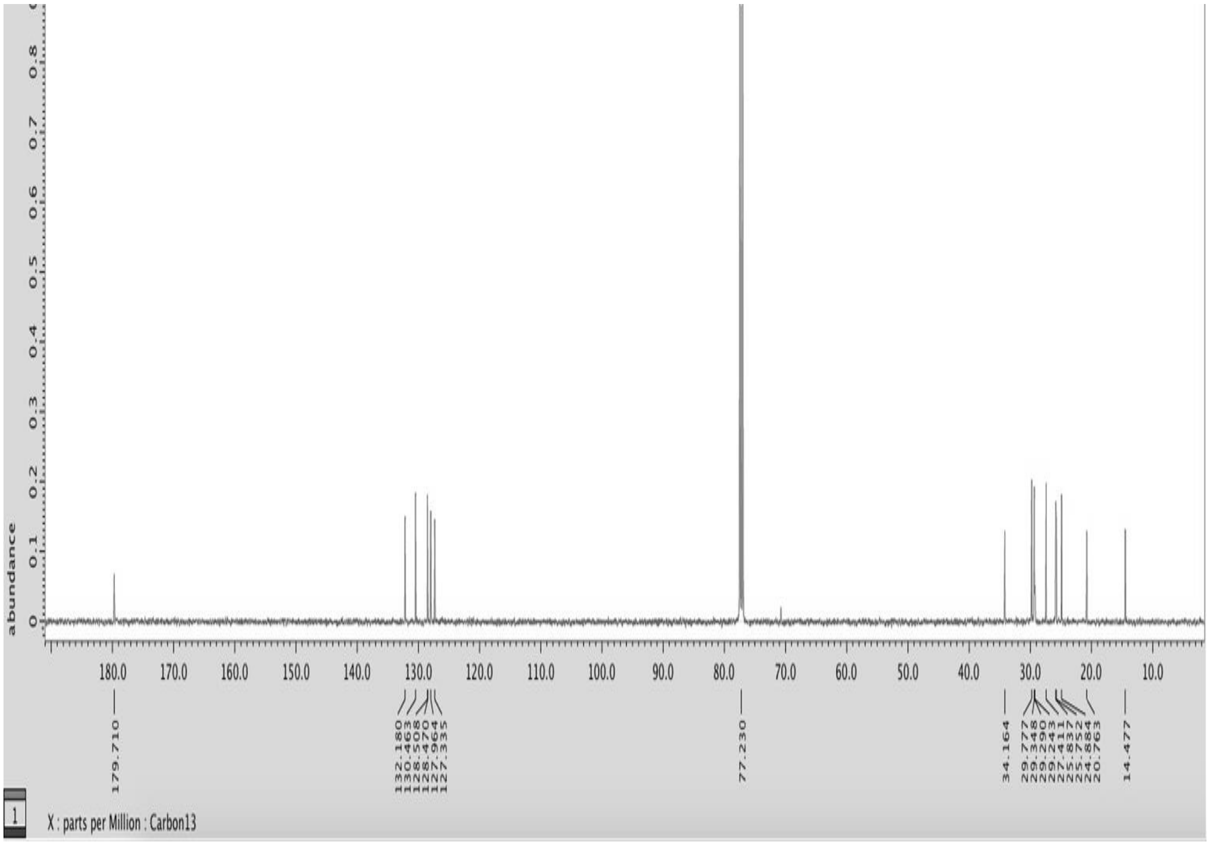

Figure S15. ^{13}C NMR of 9,12,15-Octadecatrienoic acid (**3**) [125 MHz, CDCl3].

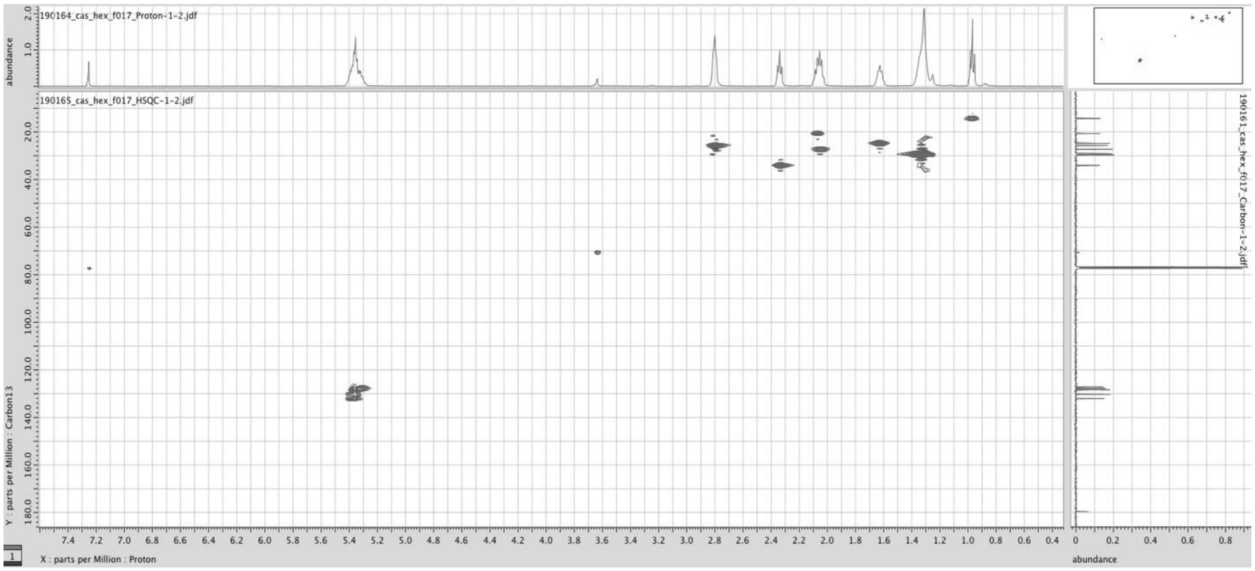

Figure S16. $^{1}H \rightarrow ^{13}C$ HSQC NMR spectrum of 9,12,15-Octadecatrienoic acid (**3**) [500 MHz, CDCl3].

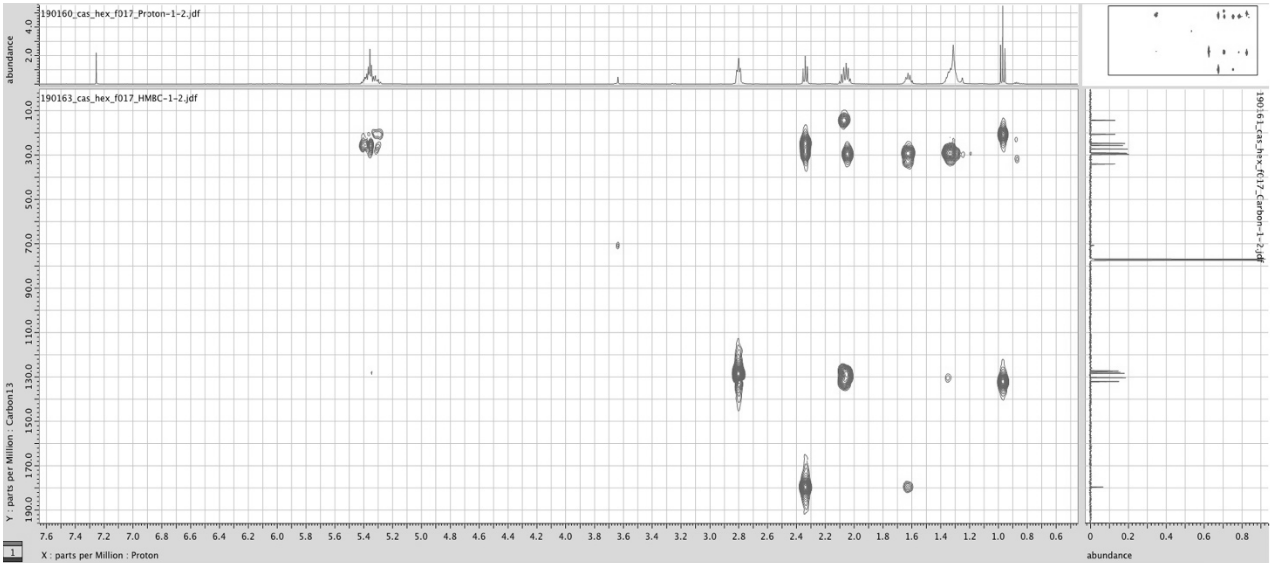

Figure S17. $^{1}H\rightarrow^{13}C$ HMBC NMR spectrum of 9,12,15-Octadecatrienoic acid (**3**) [500 MHz, CDCl3].

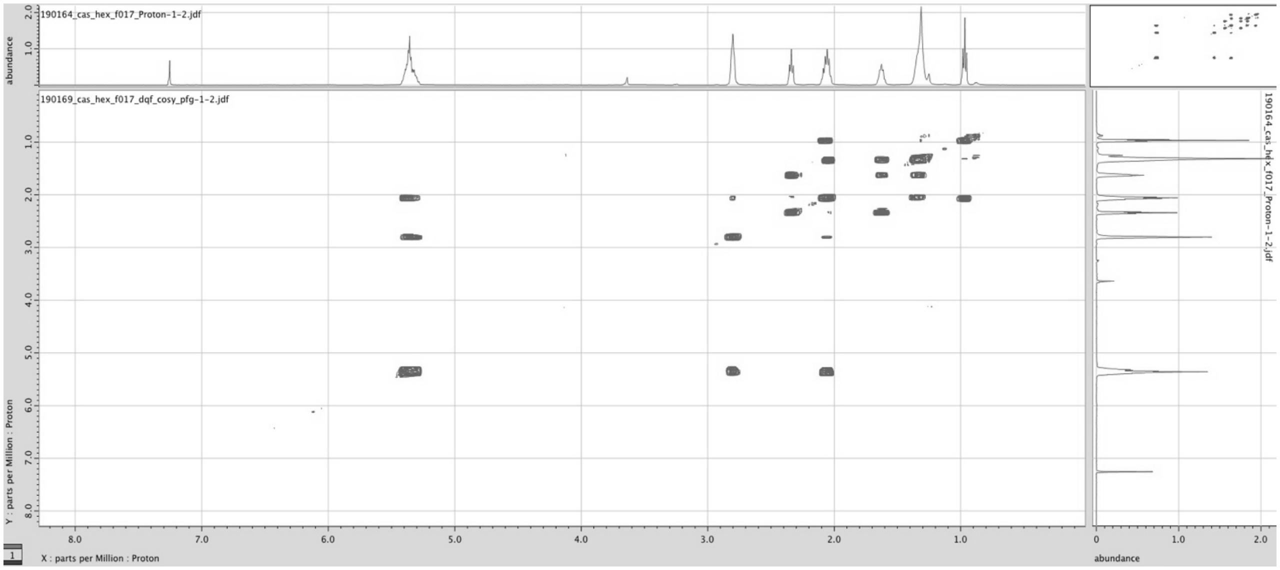

Figure S18. $^{1}H\rightarrow^{1}H$ COSY NMR spectrum of 9,12,15-Octadecatrienoic acid (**3**) [500 MHz, CDCl3].

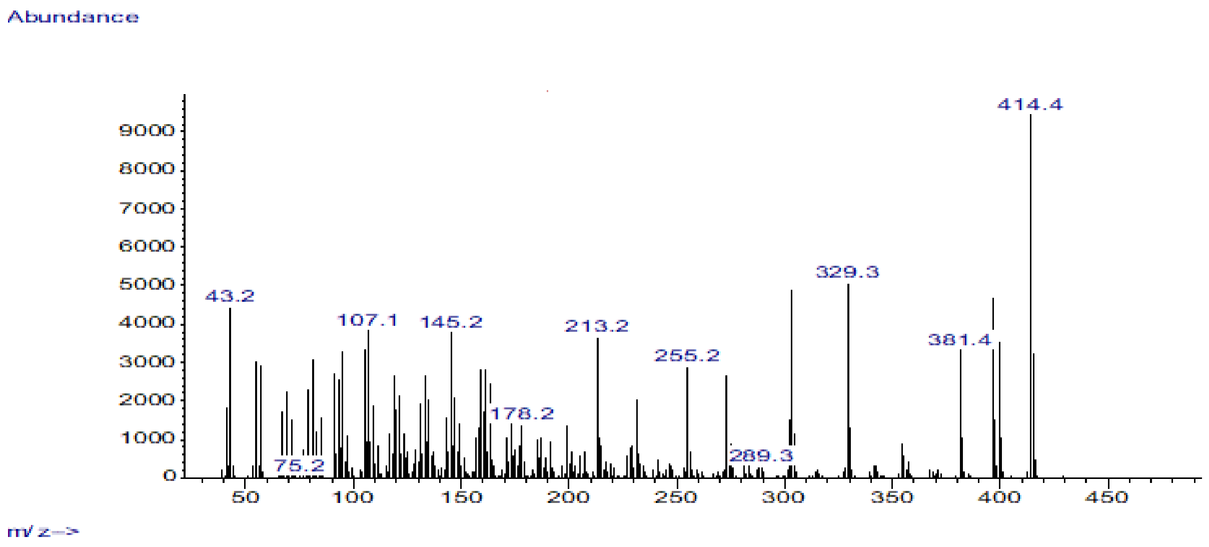

Figure S19. Mass spectrum of *β*-sitosterol **(4)**.

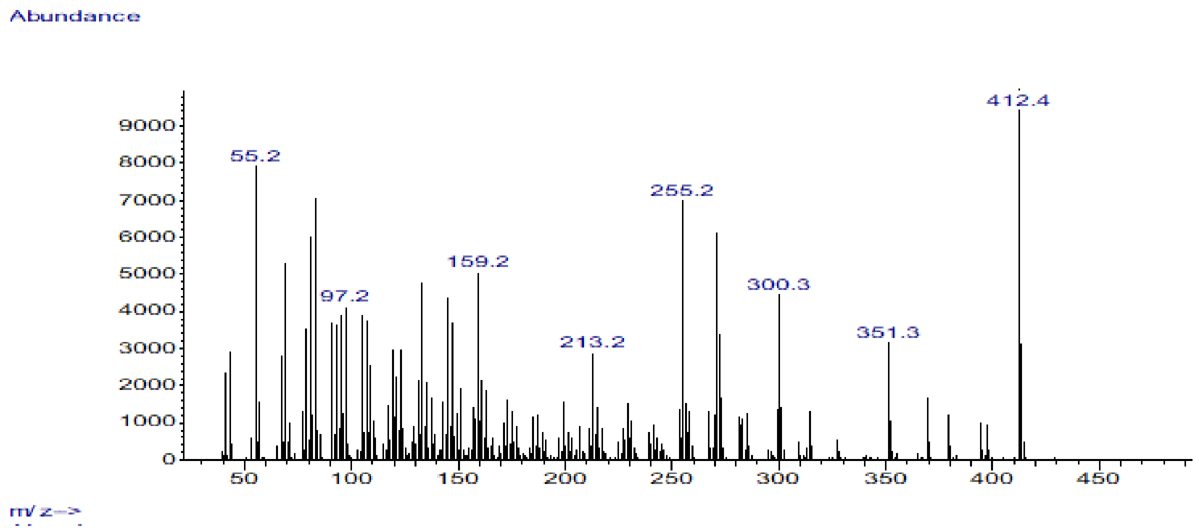

Figure S20. Mass spectrum of stigmasterol **(5).**

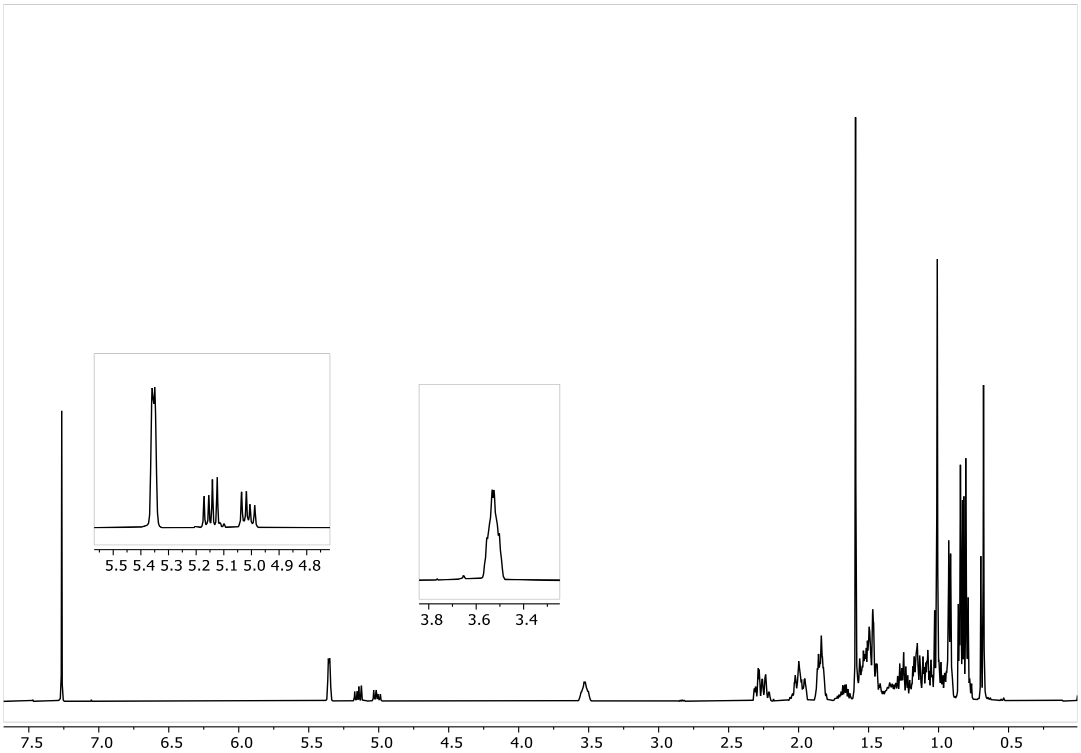

Figure S21. ^{1}H NMR spectrum of the mixture of β-sitosterol **(4)** and stigmasterol **(5)** [700 MHz, $CDCl_3$].

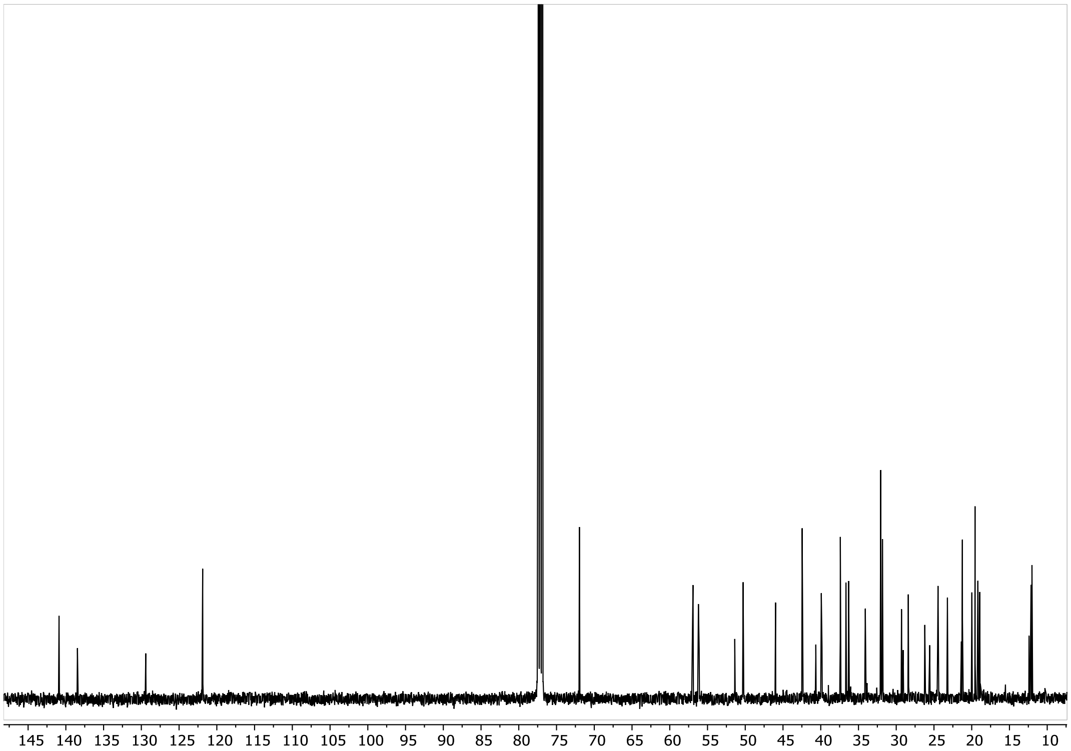

Figure S22. ^{13}C NMR spectrum of the mixture of β-sitosterol **(4)** and stigmasterol **(5)**[175 MHz, $CDCl_3$].

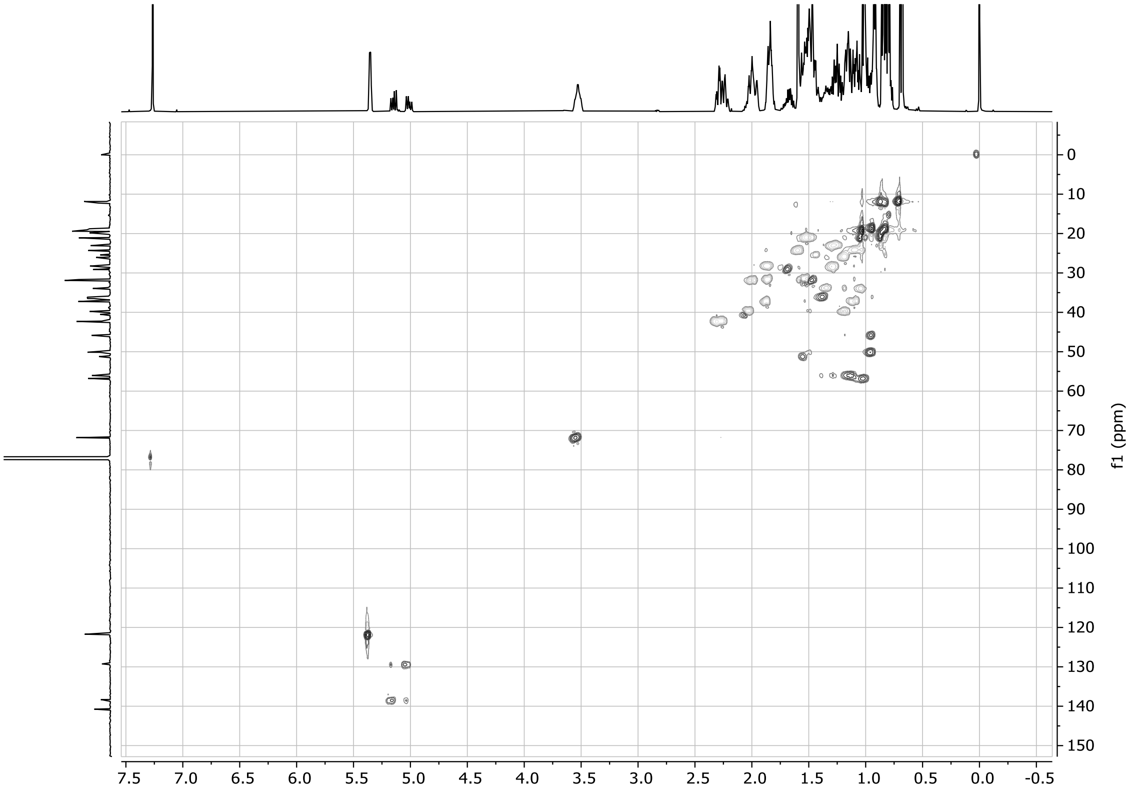

Figure S23. $^{1}H \rightarrow ^{13}C$ HSQC NMR spectrum of β-sitosterol and stigmasterol [700 MHz, $CDCl_3$].

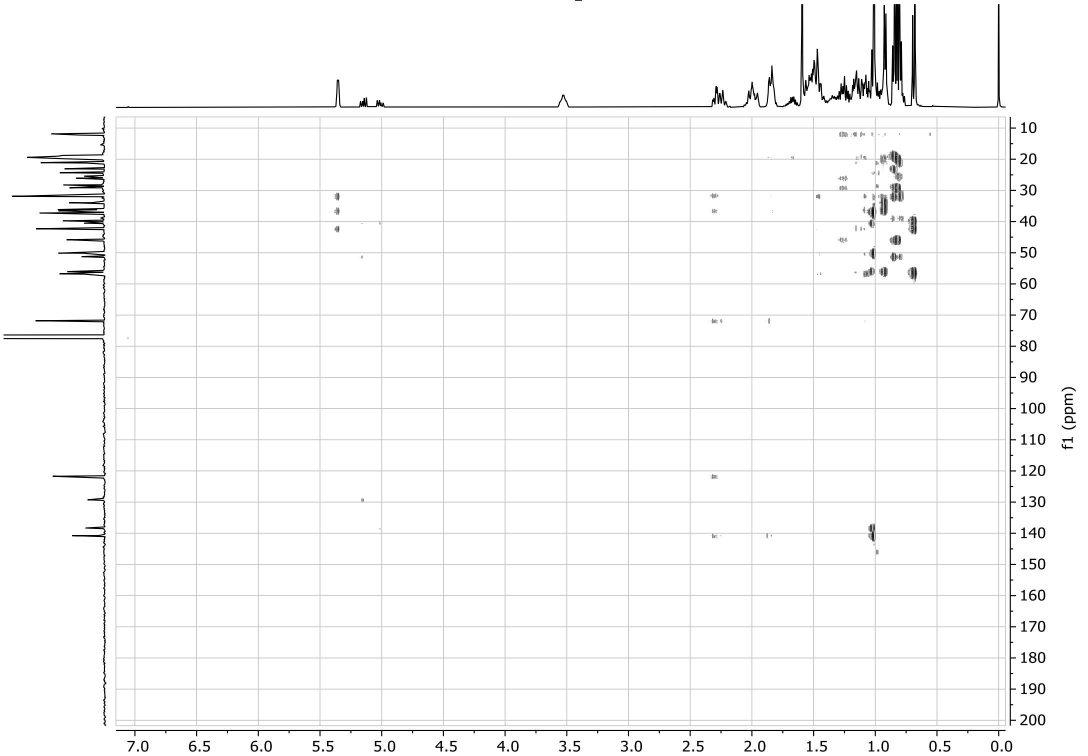

Figure S24. $^{1}H \rightarrow ^{13}C$ HMBC NMR spectrum of β-sitosterol **(4)** and stigmasterol **(5)** [700 MHz, $CDCl_3$].

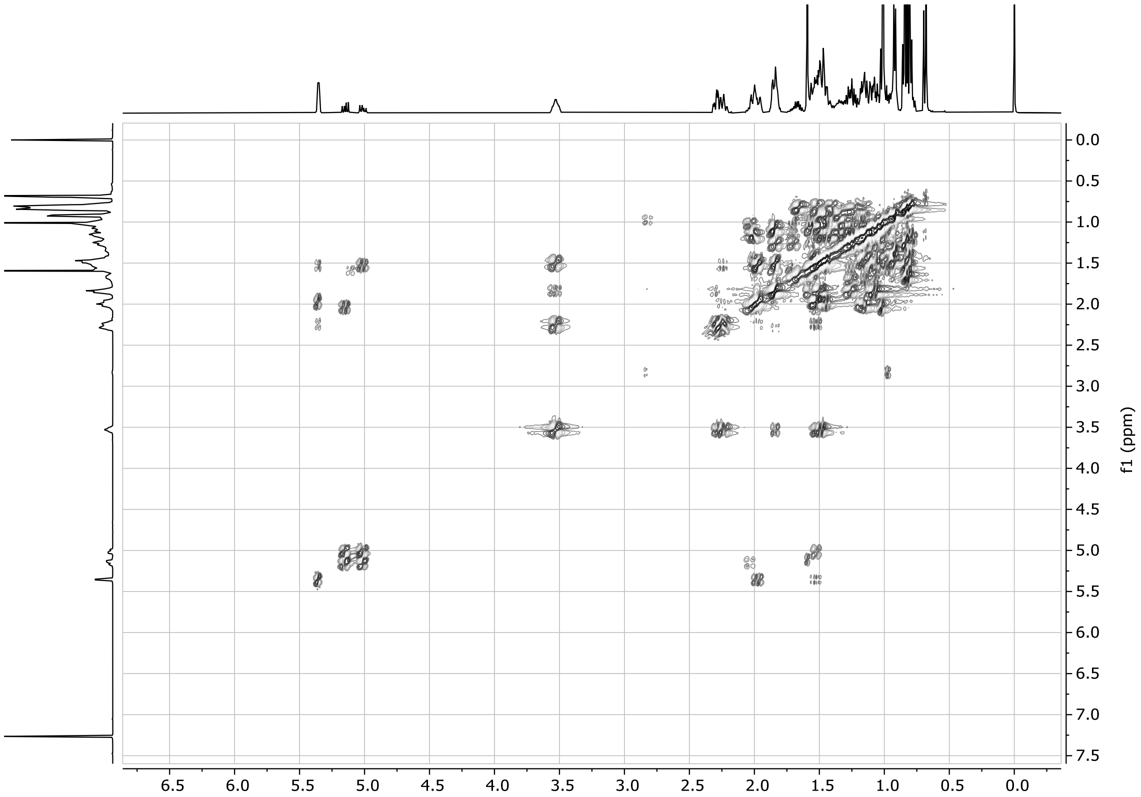

Figure S25.^{1}H→^{1}H COSY NMR spectrum of β-sitosterol **(4)** and stigmasterol **(5)** [700 MHz, $CDCl_3$].

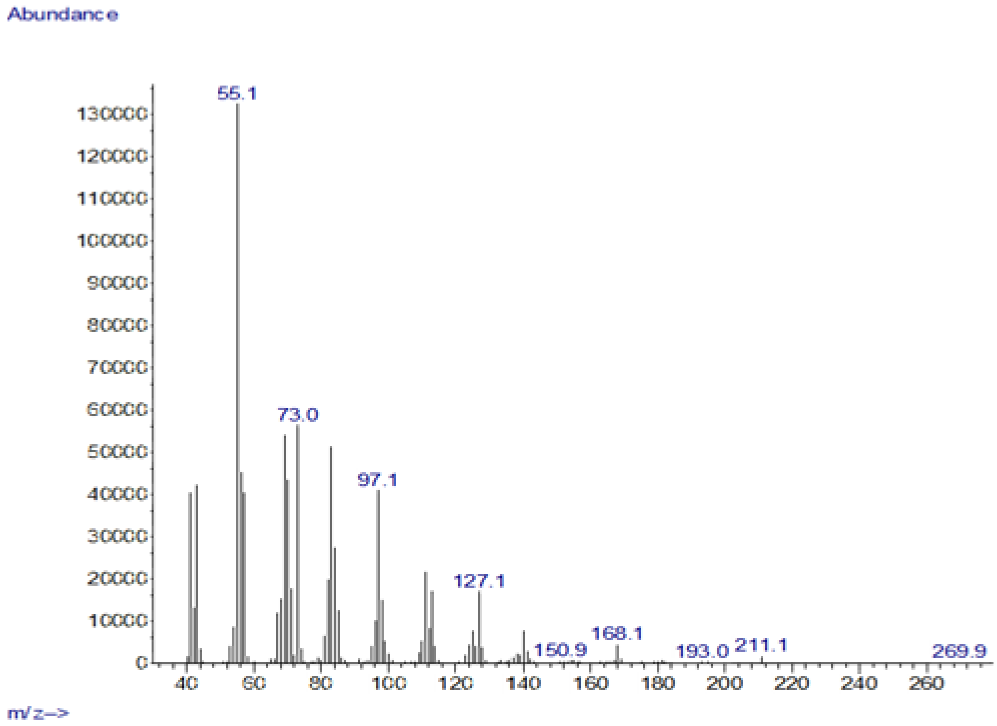

Figure S26. Mass spectrum of 1-octadecanol **(6)**.

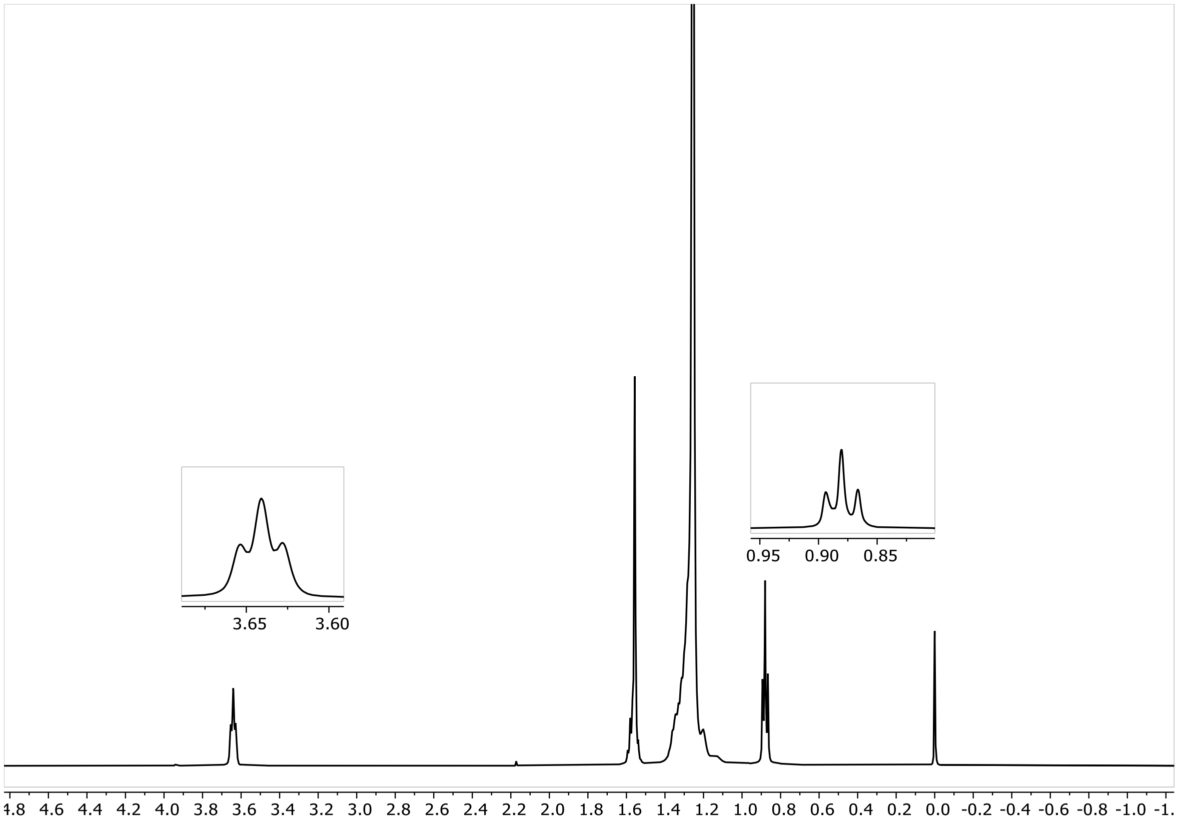

Figure S27. ^{1}H-NMR of 1-octadecanol **(6)** [700 MHz, $CDCl_3$].

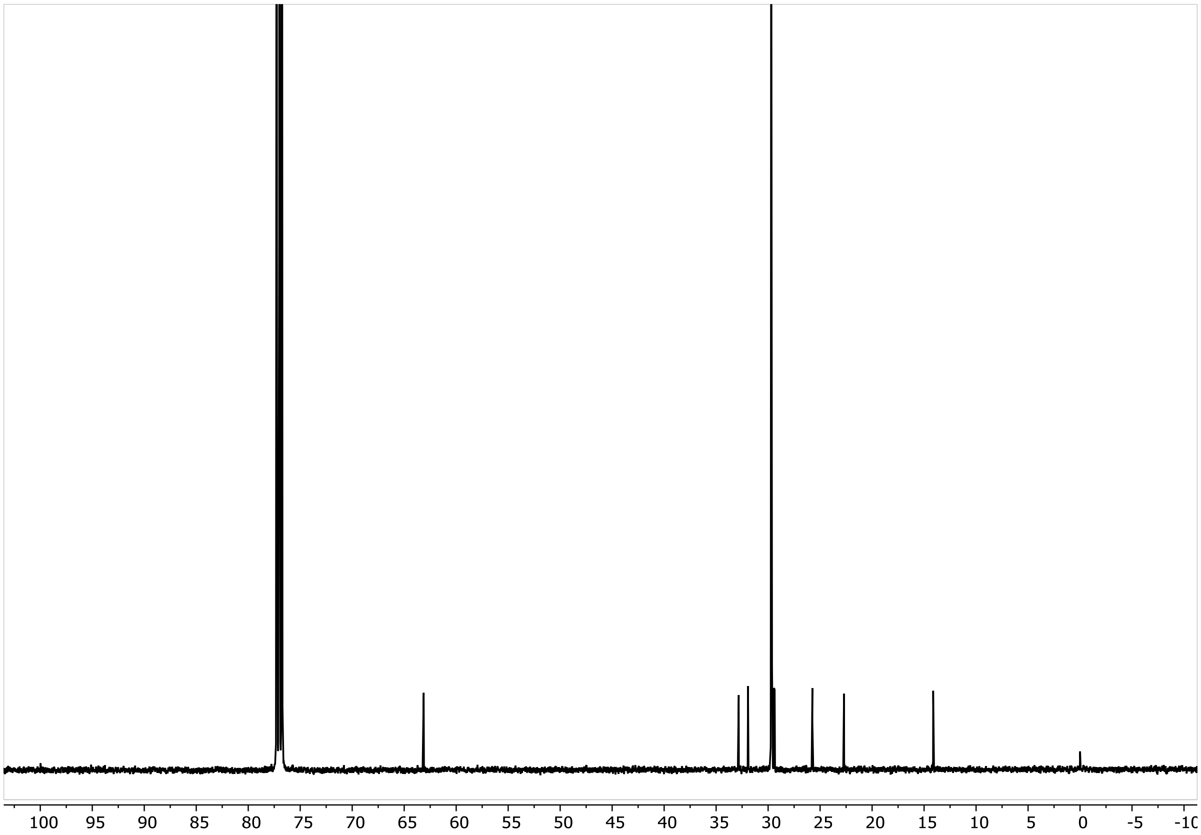

Figure S28. ^{13}C NMR spectrum of 1-octadecanol **(6)** [175 MHz, $CDCl_3$].

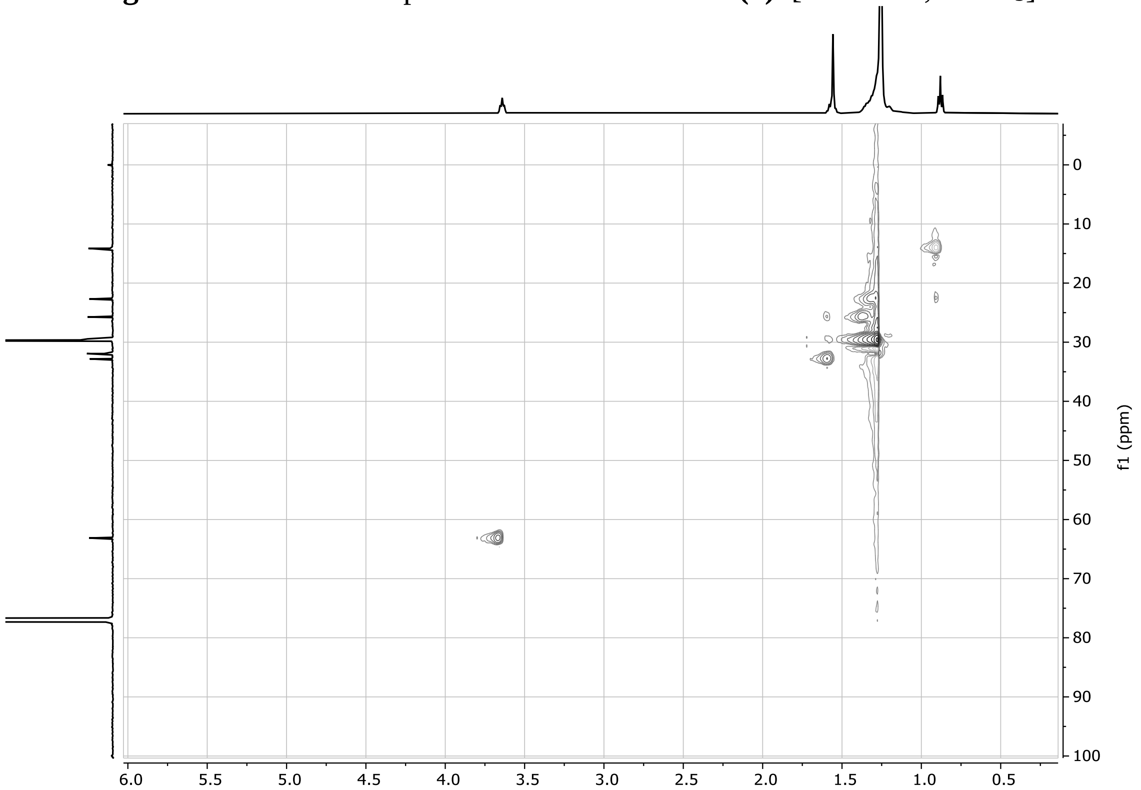

Figure S29. $^{1}H \rightarrow ^{13}C$ HSQC NMR spectrum of 1-octadecanol **(6)** [700 MHz, $CDCl_3$].

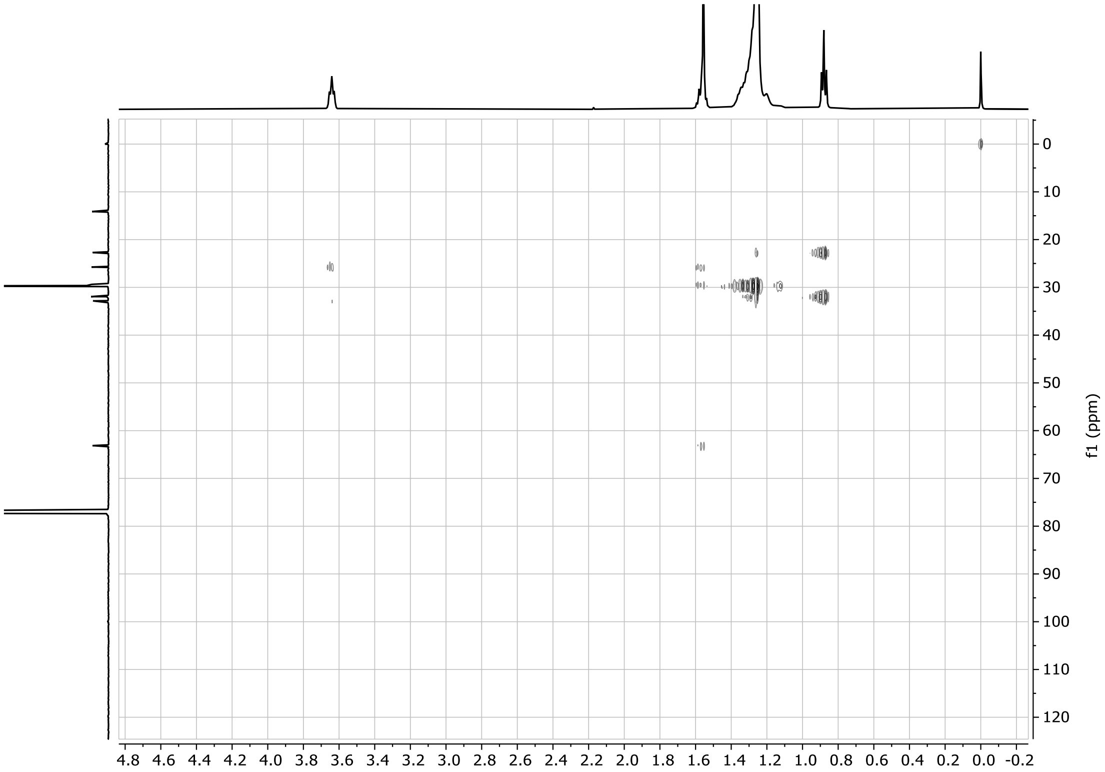

Figure S30. $^{1}H \rightarrow ^{13}C$ HMBC NMR spectrum of 1-octadecanol **(6)** [700 MHz, $CDCl_3$].

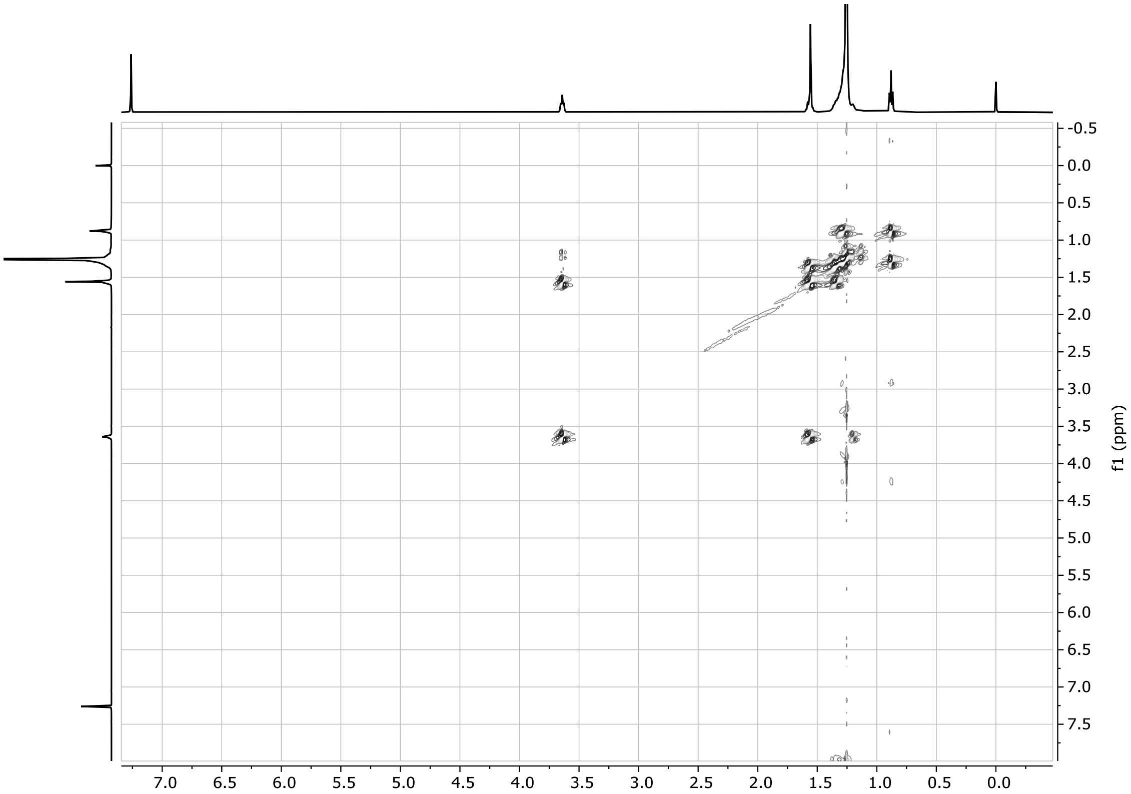

Figure S31. $^{1}H \rightarrow ^{1}H$ COSY NMR spectrum of 1-octadecanol **(6)** [700 MHz, $CDCl_3$].
